# A Dual-Color Far-Red to Near-Infrared Firefly Luciferin Analogue Designed for Multiparametric Bioluminescence Imaging**

**DOI:** 10.1002/anie.201405955

**Published:** 2014-09-29

**Authors:** Amit P Jathoul, Helen Grounds, James C Anderson, Martin A Pule

**Affiliations:** Department of Haematology, UCL Cancer Institute and NIHR University College London Hospitals Biomedical Research CentreLondon, WC1E 6BT (UK); Department of Chemistry, University College LondonLondon, WC1E 6BT (UK)

**Keywords:** imaging agents, luciferase, luminescence, multiparametric imaging, structure–activity relationships

## Abstract

Red-shifted bioluminescent emitters allow improved in vivo tissue penetration and signal quantification, and have led to the development of beetle luciferin analogues that elicit red-shifted bioluminescence with firefly luciferase (Fluc). However, unlike natural luciferin, none have been shown to emit different colors with different luciferases. We have synthesized and tested the first dual-color, far-red to near-infrared (nIR) emitting analogue of beetle luciferin, which, akin to natural luciferin, exhibits pH dependent fluorescence spectra and emits bioluminescence of different colors with different engineered Fluc enzymes. Our analogue produces different far-red to nIR emission maxima up to *λ*_max_=706 nm with different Fluc mutants. This emission is the most red-shifted bioluminescence reported without using a resonance energy transfer acceptor. This improvement should allow tissues to be more effectively probed using multiparametric deep-tissue bioluminescence imaging.

Bioluminescence imaging (BLI) has revolutionized molecular genetic imaging in biomedical research as a cheap and easy means to longitudinally image the genetic behavior of life and disease processes in whole mammals.[1–4] As they produce the brightest form of bioluminescence,[5] genes from coleopterans are commonly used to localize, track, and quantify cells and molecular or functional events in vivo.[6–8] In a well-studied reaction,[9] beetle luciferin (**1**, Figure 1 a) is adenylated by firefly luciferase (Fluc) and this reacts with molecular oxygen to produce an excited state species, oxyluciferin* (**2**), which decays to release a photon with a high quantum yield (*λ*_max_=558 nm).[5] However, absorption of visible light by hemoglobin (Hb) and melanin restricts image resolution and signal penetration at this wavelength. Between *λ*=600–800 nm, the absorption of light by Hb decreases by a factor of approximately 50, resulting in less attenuation of red light. This wavelength range is within what is termed the “bio-optical window” and there has been much focus on engineering red-shifted Fluc enzymes that have maximum emission wavelengths in this range,[10–15] but these have peaked at wavelengths less than *λ*=645 nm.

**Figure 1:**
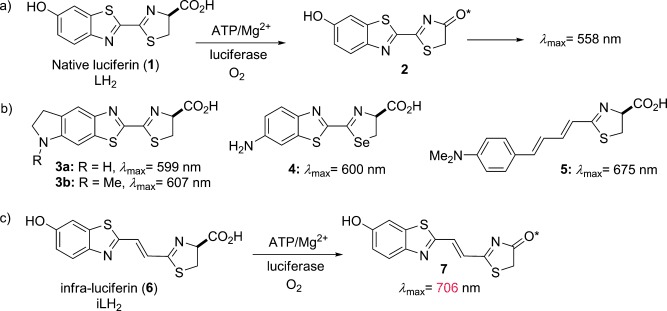
a) Bioluminescence of luciferin (1) catalyzed by luciferase. b) Structures of red-shifted bioluminescent amino-luciferin analogues. c) New luciferin analogue iLH_2_ 6 that exhibits near-infrared bioluminescence with mutant Fluc. ATP=adenosine triphosphate.

The most red-shifted luciferin analogues to date[16] are based upon amino derivatives (Figure 1 b), for example cyclic aminoluciferin (**3 a**: *λ*_max_=599 nm; **3 b**: *λ*_max_=607 nm),[17] seleno-d-aminoluciferin (**4**: *λ*_max_=600 nm),[18] and a rationally designed 4-(dimethylamino)phenyl derivative conjugated to a thiazoline group (**5**: *λ*_max_=675 nm).[19] In particular cyclic aminoluciferin derivative **3 a** has been shown to give improved bioluminescence imaging compared to luciferin (LH_2_; **1**) at dilute concentrations where the intracellular concentration of the luciferin or analogue is limiting.[20] Near-infrared emission has been detected with an aminoluciferin Cy5 conjugate, but this is due to bioluminescence resonance energy transfer (BRET),[21] meaning that the conjugate cannot be used for multiparametric imaging.

To date, despite a red-shift in emission, no analogues have been reported with the other desirable properties of LH_2_ **1**, such as a high quantum yield and the ability to produce more than one color with different Fluc mutants. Considering this, and the likely mechanisms of color tuning in Fluc bioluminescence, we describe the design, synthesis, and in vitro and in vivo testing of the first far-red to nIR multicolor-emitting analogue, which can produce the most red-shifted form of true bioluminescence reported to date. Additionally, our far-red-shifted analogue infra-luciferin (**6**, iLH_2_; Figure 1 c) produces distinct bioluminescent colors with different enzymes akin to native luciferin, and could be of great benefit to multiparametric deep-tissue and tomographic bioluminescence in vivo imaging.

Despite a number of theories, the exact mechanism regulating color tuning in Fluc bioluminescence has not been solved.[11–13] Current measurements and calculations suggest that color modulation is due to perturbing interactions in the microenvironment surrounding the anionic phenolate of excited-state oxyluciferin (**2**) in the Fluc active site.[22–28] Additionally, π–π overlap between the benzothiazole and thiazolone heterocycles in **2** also appears to be important.[29–31] Maki and co-workers demonstrated the importance of extended π-conjugation in luciferin derivatives which led to the development of **5**.[19,32] In our design we proposed that increasing the conjugation of LH_2_ **1**, and thus **2**, by addition of an alkene linker between the benzothiazole and thiazoline fragments would lead to a red-shifted luciferin analogue (**6**, Figure 1 c) that would be amenable to color modulation with different Fluc mutants. Extended conjugation should reduce the HOMO–LUMO energy gap in the light-emitting phenolate of **7**, which would lead to red-shifting of the emitted light. Our design, in contrast to other established red-shifted luciferin analogues (Figure 1), retained the 6′-hydroxy group. This design was chosen in an attempt to capitalize on the microenvironment effect of different Fluc mutants to generate different bioluminescence emission wavelengths that are essential for multiparametric imaging. We also believed that the increase in the overall shape of the molecule by only one alkene unit may be tolerated by Fluc mutants to facilitate multiwavelength emission.

The molecule iLH_2_ **6** was synthesized in 10 steps from commercially available starting materials (Scheme 1). During the synthesis we found that once the thiazoline ring had been formed the molecule was incredibly sensitive to epimerization next to the carboxy group. Both the methyl and ethyl ester of iLH_2_ **6** could be isolated in enantiopure form, but isolation of the free acid after saponification was found to be extremely difficult with epimerization and formation of the thiazole detected. To maximize light output we decided to test the enantiopure esters in vitro and in vivo as it has been shown that esters of LH_2_ **1** are active in live cells and living mice,[33,34] as they are saponified by esterases. We also synthesized the Maki analogue **5**,[19] the most red-shifted bioluminescent analogue reported to date, to compare its properties to iLH_2_ **6**.

**Scheme 1:**
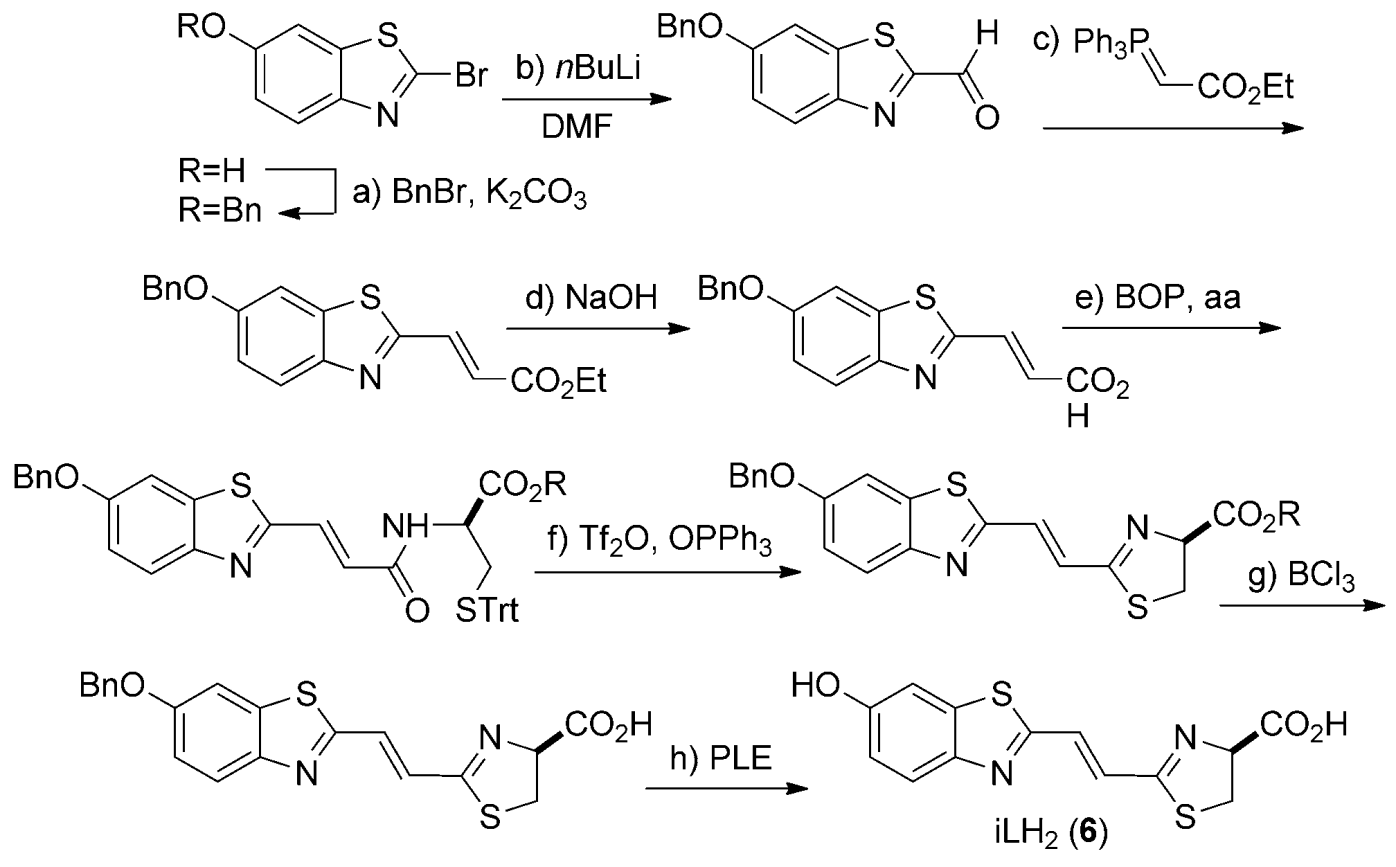
Synthesis of infra-luciferin 6. a) BnBr (1.2 equiv), K_2_CO_3_ (2.8 equiv), acetone, room temperature, 16 h, 85 %; b) *n*BuLi (1.93 m in hexanes, 1.1 equiv), THF, −78 °C, 15 min then DMF (4.1 equiv), 1 h, 96 %; c) (Carbethoxymethylene)triphenylphosphorane (3 equiv), PhMe, reflux, 3 h, 92 %; d) NaOH (1 m), *i*PrOH, 16 h, quantitative yield; e) Et_3_N (2.4 equiv), DMF, amino acid (aa; 1.2 equiv), 0 °C then BOP (1.2 equiv) in CH_2_Cl_2_, 2 h (R=Me, 80 %, R=Et, 82 %); f) Ph_3_PO (1.3 equiv), Tf_2_O (2.7 equiv), CH_2_Cl_2_, 0 °C, 30 min added to benzothiazole in CH_2_Cl_2_, 0 °C, 10 min, (R=Me, 65 %, R=Et, 74 %); g) pentamethylbenzene (4.4 equiv), BCl_3_ (1 m in CH_2_Cl_2_, 3 equiv), CH_2_Cl_2_, −78 °C, (R=Me, 79 %, R=Et, 72 %); h) PLE, buffer, 37 °C, in situ. Bn=benzyl; Tf_2_O=trifluoromethanesulfonic anhydride; Trt=triphenylmethyl; BOP=(benzotriazol-1-yloxy)tris(dimethylamino)phosphonium hexafluorophosphate; PLE=pig liver esterase.

The fluorescence spectra of iLH_2_ **6** compared to LH_2_ **1** showed that at pH 7, the emission maximum of **6** was *λ*_max_=588 nm, with a red shift of 58 nm compared to **1**. Furthermore, the fluorescence excitation and emission spectra are pH dependent, as measured for **1**. In contrast, the Maki analogue **5** had pH independent fluorescence spectra, producing only one fluorescence excitation color (see Figures S1 a–c in the Supporting Information). This highlights the importance of retaining the 6′-hydroxy group for color modulation.[22–28]

In vitro bioluminescence spectra of iLH_2_ **6** ethyl ester (saponified with PLE (pig liver esterase) in situ immediately prior to use) with purified wild-type (WT) Fluc, the x5 Fluc mutant (a thermostable Fluc with similar properties to WT but with higher quantum yields),[35,36] and the x5 S284T Fluc mutant (a bright red-shifted point mutant of x5)[37,38] showed marked red-shifted peak maxima of 100 nm magnitude compared to the *λ*_max_ of each enzyme with **1** (Figure 2, Table 1). This effect is remarkable considering that these mutants were originally engineered for different emission colors with LH_2_ **1**. Our analogue showed bioluminescence with *λ*_max_=706 nm with Fluc mutant x5 S284T, significantly red-shifted compared to any reported natural or unnatural bioluminescence system. This suggests that Fluc mutants emit different color forms with **1** and **6** in line with the microenvironment effect of the Fluc active sites.[12,22–28] In contrast, bioluminescence of the Maki analogue **5** with different Fluc mutants occurred with almost identical emission wavelength maxima and only very small nonspecific shifts between Fluc mutants (Figure S2 a),[19] supporting the theory that the phenolic hydroxy group is crucial for the color-tuning mechanism of luciferin.[22–28] These results suggest that iLH_2_ **6** would be of use for in vivo multiparametric imaging, whereas the Maki analogue **5** would not be. The other red-shifted luciferin analogues (Figure 1) may also not be suitable because of the substitution of the phenolic hydroxy group for an amine.

**Figure 2:**
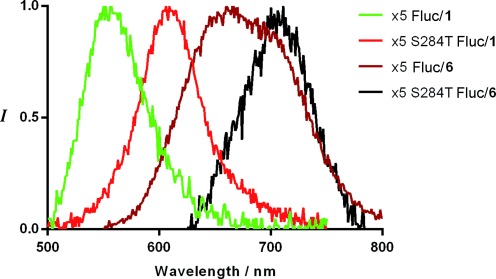
Bioluminescence spectra of native 1 and 6 with x5 and x5 S284T Fluc mutants.

**Table 1:** Bioluminescence spectral properties of 1, 5, and 6 with purified enzymes.

Fluc Mutant	Luciferin Substrate	*λ*_max_ [nm]	FWHM [nm][a]
WT Fluc	**1**	558	76
	**5**	652	76
	**6**	670	74
X5 Fluc	**1**	554	62
	**5**	652	76
	**6**	646	92
X5 S284T	**1**	605	56
	**5**	658	78
	**6**	706	81

[a] FWHM=bioluminescence full width at half maximum. See Supporting Information for experimental conditions.

The proportion of light output above *λ*=600 nm is a key determinant for transmission efficiency through mammalian tissue.[39] Bioluminescence data were acquired using different band-pass (bp) filters in a Photon Imager and showed that most light from the x5 Fluc mutant with **6** was collected through a *λ*=670–720 nm bp filter and from the x5 S284T mutant through a *λ*=700–750 nm bp filter. The proportion of light emitted over *λ*=600 nm with LH_2_ **1** was 32 % (WT Fluc), 18 % (x5 Fluc), and 68 % (x5 S284T Fluc), respectively. For iLH_2_ **6**, this increased dramatically to 95 %, 97 %, and 100 %, respectively (Figure S2 b). This should have a significant impact on the amount of transmitted light detected during in vivo imaging (see below).

To compare the in vitro specific activity of the x5 Fluc mutant with the saponified esters (PLE) of LH_2_ **1**, the Maki analogue **5**, and iLH_2_ **6**, data was acquired using an IVIS 200 instrument—Caliper Life Sciences, USA (Table 2). With purified enzymes, a difference of approximately 100-fold was seen in the specific activity of x5 Fluc from LH_2_ **1** and iLH_2_ **6**, whereas **5** displayed a 3-fold lower specific activity than **1**. Apparent kinetic parameters indicate that the affinity (*K*_m_) of x5 Fluc for infra-luciferin (**6**) are more similar to LH_2_ **1** than **5**, though the turnover (*k*_cat_), and therefore the overall catalytic efficiency (*k*_cat_ *K*_m_^−1^), of **6** was approximately 285-fold lower than **1**, compared to 25-fold lower for **5**. A similar magnitude of a decrease in the *k*_cat_ value has been reported for commercially available thermostable luciferase mutants compared to WT Fluc with **1**.[36] The full width at half maximum (FWHM) of emission of both x5 Fluc and x5 S284T are markedly wider with infra-luciferin (**6**) than with LH_2_ **1**, indicating less emitter specificity of the x5 mutant framework (Table 1). It may be possible to engineer Fluc mutants with enhanced activities, kinetic parameters, and/or emitter specificity with infra-luciferin (**6**).[15,35,40,41] Despite **6** having lower activity, the benefit of **6** is that it exhibits near-infrared bioluminescence that is susceptible to color modulation by mutated Fluc enzymes, unlike **5**, and thus shows promise for multiparametric imaging.

**Table 2:** In vitro activity and apparent kinetic parameters of the x5 Fluc mutant with the saponified esters of 1, 5, and 6.[a]

Substrate	Apparent *K*_m_ [μm]	Apparent *k*_cat_ [RLU s^−1^×10^15^]	Apparent *k*_cat_/*K*_m_ [s^−1^ μm^−1^×10^15^]	Specific Activity/cpm [cm^−2^ mg^−1^×10^15^]
**1** Et Ester	2.0	40.0	200	60.9
**5** Me Ester	16.7	13.3	8.0	19.7
**6** Me Ester	6.0	0.4	0.7	0.6

[a] Data recorded in triplicate. See Supporting Information for experimental conditions. cpm=counts per minute. RLU=relative light units.

Cells transduced with Fluc readily showed bioluminescence activity upon treatment with iLH_2_ **6**. With this in mind, we established a number of mouse models of cancer with the aim of detecting and imaging these using iLH_2_ **6** methyl ester and comparing in vivo light yields to native luciferin **1** (Figure 3 a–c).[42] A subcutaneous (sc) model was induced by sc injection of 5×10^6^ LS174T cells (colon carcinoma cell line) expressing WT Fluc into Nude (MFI NuNu) mice (Figure 3 a). After four days post-inoculation, mice were intraperitoneally (ip) injected with LH_2_ **1** ethyl ester (2 mg) or iLH_2_ **6** methyl ester and imaged in the Photon Imager. Light emission was readily apparent in mice administered with iLH_2_ **6** methyl ester. In vivo spectra showed that the maximum emission wavelength for WT Fluc with LH_2_ **1** is within the *λ*=590–640 nm bp filter, likely as a result of the bathochromic shift of WT Fluc at physiological temperature and also attenuation of the true spectrum by Hb in mouse tissues (Figure S3).[39] However, the in vivo spectrum of WT Fluc with iLH_2_ **6** displays a *λ*_max_ in the *λ*=700–750 nm bp filter and its shape appears much less attenuated by mouse tissues. We also found iLH_2_ **6** methyl ester was active and detectable in a systemic lymphoma mouse model of cancer (Figure 3 b). In this case, the same mice were imaged on consecutive days with LH_2_ **1** ethyl ester or iLH_2_ **6** methyl ester. One minute background luminescence images were acquired prior to imaging to ensure there was no remaining activity from previous sessions. These images (Figure 3 b) show that **6** gives a more even intensity across the whole animal, likely because of the consequence of less attenuation by mouse tissues due to the higher proportion of light output above *λ*=600 nm (Figure S4). Finally, iLH_2_ **6** was employed to image liver metastases in an orthotopic model in nude mice (Figure 3 c, Figure S5), a particularly challenging organ to get meaningful data from because of blood and tissue attenuation. In this case, mice were imaged 15 minutes after ip injection of iLH_2_ **6** methyl ester (4 mg), or with LH_2_ **1** potassium salt (2 mg), also given ip. The relative in vivo light yield for **1** is identical to those measured for the methyl ester, showing saponification is complete in the mouse.[33,34] After just 15 seconds, the image acquisitions (Figure 3 c) show that **6** detailed the non-uniform nature of the metastatic tumor burden whilst images with **1** neared saturation and showed little definition because of its intensity.

**Figure 3:**
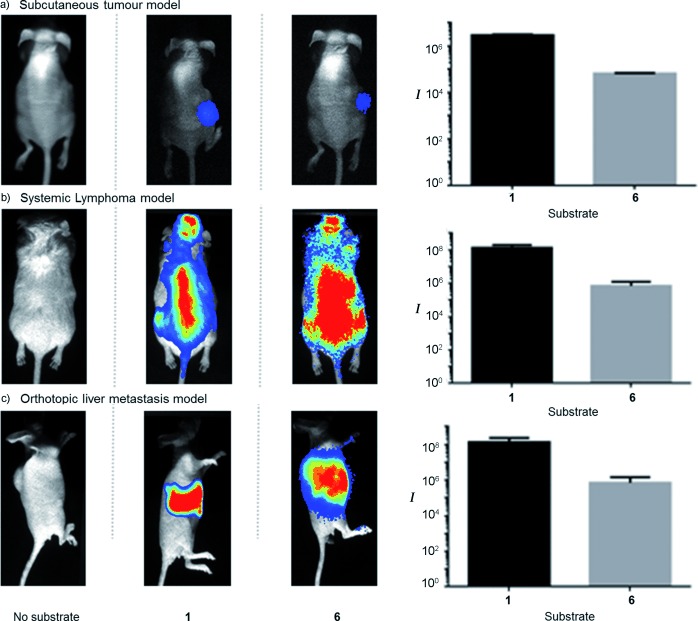
In vivo imaging with 6 in mouse cancer models expressing firefly luciferase (Fluc). Left column of images: no substrate; middle column: mice imaged with LH_2_; right column: mice imaged with iLH_2_ Me ester. Inset graphs show the relative in vivo light yields from mice with different substrates and imaged for equivalent times. In vivo spectra are displayed in Figure S4 in the Supporting Information.

Over the same time interval there was an average of 200 times less light from **6** compared to **1**. Ironically, being bright does not appear to be the absolute requirement for better penetration and resolution because of a reduced signal to noise effect caused by light scattering. Scatter of light is proportional to the reciprocal of wavelength to the fourth power and this leads to enhanced signal penetration of iLH_2_ **6** through blood when compared to luciferin (Figure 4).

**Figure 4:**
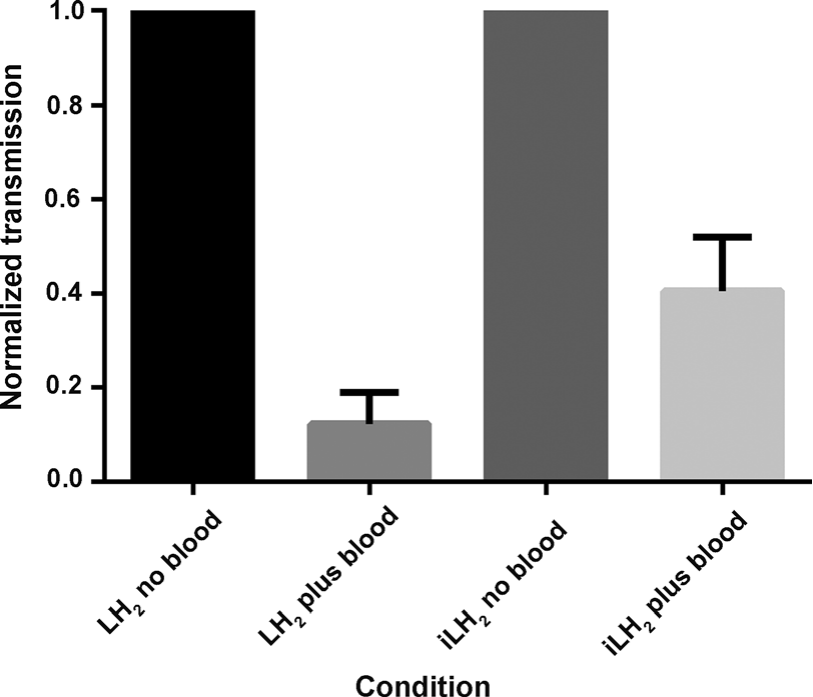
Histogram showing the increased penetration of iLH_2_ 6 emission through blood compared to LH_2_ 1.

Firefly luciferase (Fluc) produces true near-infrared bioluminescence with iLH_2_ **6**, and the emission color can be tuned with different Fluc mutants in the wavelength range of the bio-optical window of mammalian tissues, currently up to a maximum of *λ*=706 nm. As 95 % of the emitted light of iLH_2_ **6** with Fluc has an emission maximum greater than *λ*=600 nm (Figure S2 b), there is less attenuation in blood (Figure 4) and in vivo (Figure 3 c) than with LH_2_ **1**. These results suggest that this bright, red-shifted form of bioluminescence has potential for deep-tissue multiparametric BLI and could provide a more detailed assessment of in vivo cellular and molecular processes. We are in the process of testing a number of further analogue designs and are exploring the possibility of creating Fluc mutants with enhanced activity with iLH_2_ **6**.

Please note: Minor changes have been made to this manuscript since its publication in *Angewandte Chemie* Early View. The Editor.
