# Supplementary material for: A Dual-Color Far-Red to Near-Infrared Firefly Luciferin Analogue Designed for Multiparametric Bioluminescence Imaging
Source: Angew Chem Int Ed Engl. 2014 Sep 29;53(48):13059–63. doi: 10.1002/anie.201405955 (PMC4501308; doi:10.1002/anie.201405955)
Supplement: Supplementary file 1 [file anie0053-13059-sd1.pdf]

Angewandte Chemie
Eine Zeitschrift der Gesellschaft Deutscher Chemiker

Supporting Information

© Wiley-VCH 2014

69451 Weinheim, Germany

A Dual-Color Far-Red to Near-Infrared Firefly Luciferin Analogue Designed for Multiparametric Bioluminescence Imaging**

Amit P. Jathoul, Helen Grounds, James C. Anderson, and Martin A. Pule**

anie_201405955_sm_miscellaneous_information.pdf

Supplementary Information

Table of Contents

Biological methods:

Construction and purification of Fluc enzymes

Spectrofluorometry

In vitro characterisation of bioluminescence properties

Production of human retrovirus, transduction and maintenance of cell lines

In vivo bioluminescence imaging and acquisition of bioluminescence spectra

Supplementary Figure S1a

Supplementary Figure S1b

Supplementary Figure S1c

Supplementary Figure S2a

Supplementary Figure S2b

Supplementary Figure S3

Supplementary Figure S4

Supplementary Figure S5

General Experimental

Synthetic Procedures

^{1}H and ^{13}C NMR Spectra

Supplementary References

BIOLOGICAL METHODS

Construction and purification of Fluc enzymes. Vectors encoding a 10x N-terminal histidine-tagged thermostable x5 Fluc (pET16bx5), and the wild-type *Photinus pyralis* Fluc (pPW601L) were kindly donated by Drs. Laurence Tisi (Lumora LTD, Ely, Cambridgeshire, UK) and Peter White (Dstl, Porton Down, UK), respectively. Firstly, the WT gene was shuttled into pET16b by BamHI/ NcoI digestion and subcloning, and was sequenced. Site-directed mutagenesis (SDM) was carried out according to manufacturer's protocol (Quikchange^{TM}, Stratagene Corp., CA, USA) to introduce the mutation S284T into x5 Fluc. Over-expression, protein purification by NiNTA chromatography, SDS-PAGE and protein assays were carried out as detailed previously.[1]

Spectrofluorometry. Absorbance and fluorescence spectra were acquired in Thermo Flash (Thermo Fisher Scientific, MA, USA) by 1:10 dilution of free acids of luciferin (**1**), our analogue (**6**) and the Maki red analogue (**5**) in TEM buffer at pH 7.8 at room temperature. See Supplementary Figure S2 legend for details. To measure pH dependence of fluorescence spectra compounds were diluted in TEM buffers prepared between pH 6-9.

In vitro characterisation of bioluminescence properties. In vitro data was obtained by saponifying the esters using Porcine Liver Esterase (PLE – Sigma Aldrich, MO, USA) for at least 15 min prior to the addition of luciferase, without isolation of the free acid. TEM buffer (100 mM Tris-acetate, 2 mM ethylenediaminetetraacetic acid (EDTA) and 10 mM magnesium sulfate ($MgSO_4$)) was pH adjusted at room temperature (RT) with 1-10 M sodium hydroxide or 6-10 M acetic acid. Bioluminescence spectra were acquired using a Cary Eclipse spectrophotometer (Agilent Technologies, CA, USA) using PMT gains of 800-1000 V and emission slits of 20 nm and 100 ms gating. Signal averaging was carried out if required (Figure 2) and spectra were corrected for variation in photomultiplier tube spectral sensitivity by calibrating with lucifer yellow (Sigma, MA, USA) and 2-[4-(Dimethylamino)styryl]-1-methylpyridinium iodide (ASP+) (Sigma, MA, USA) dyes, and using the known corrected spectra from Molecular Probes.

Figure 2, Table 1: To initiate bioluminescence, 50 μL of 0.1 mg/mL **1** or 1 mg/mL of **5** or **6** and 20 μL 20mM ATP was added to 20 μL 7μM solutions of Fluc in cold TEM buffer. Specific activities of enzymes were calculated by integrating light emission from 1 mM substrates, 5 mM ATP and 0.5 μM Fluc in 100 uL TEM at room temperature and using no

filter for 2 min in the Photon imager device (Biospace Labs, Paris, France). FWHM: bioluminescence Full-width half-maximum. Details as in Table 2. Error +/-2 nm.

Table 2: Compounds **1** Et ester, **5** Me ester and **6** Me ester (4mg) were dissolved in 400µl ethanol before 1.6ml 10mM ammonium bicarbonate containing 2.5mg/ml porcine liver esterase was added and incubated at 37°C at 200rpm for 19hrs. Then dilutions of 10µl of 0.64µM – 1mM of esters were added to 40µl of 5mM ATP containing 0.5µM pure x5 Fluc in TEM buffer to initiate bioluminescence. Light emission over 2s was captured in an IVIS 200 imager (Caliper, USA) and kinetic parameters were calculated using the Hanes-Woolf plot.[2] Specific activities of **1**, **5** and **6** esters with purified enzymes: to capture specific activities of enzymes with substrates, 40 µL 0.5 µM x5 Fluc in 5 mM TEM buffered ATP was added to saponified esters and light emission was captured for 2s using the IVIS Imager.

Production of human retrovirus, transduction and maintenance of cell lines. Retroviral vector was generated by co-transfecting 293T cells with vector plasmid, along with RDF plasmid which supplied the RD114 envelope as described previously.[3] Cell lines were either purchased from ATCC, or provided by the UCL Cancer Institute cell bank. Flow cytometry was carried out using anti-Myc primary (Invitrogen Life Technologies, CA, USA) and anti-Fc DyeLight 647 (Jackson ImmunoResearch, PA, USA) secondary conjugated antibodies in a CyAn instrument (Beckman Coulter, Inc., Brea, CA, USA). The percentage of stained cells (expressing the Myc tag) was used to ascertain the percentage transduction. The mean fluorescence intensity of the DY 647 antibody was used as a measure of expression level of the gene cassette. Transduction of 3 x 10^5 Raji cells with 1.5 mL of viral supernatant was carried out by using retronectin (Takara Bio Inc., Shiga, Japan) according to manufacturer's instructions. Cells were cultured at 37 °C with 5% carbon dioxide in RPMI1640 with 10% foetal calf serum (FCS) and glutamine (GlutaMAX^{TM}, Invitrogen Life Technologies, CA, USA). After 2 d, cells were harvested and analysed by flow cytometry. For LS174T cells, 1.5 x 10^6 cells were cultured overnight in a 6-well plate in Iscoves Modified Dulbeccos Medium (IMDM, Lonza, Basel, Switzerland) with 10% FCS and glutamine and 1.5 mL of virus was added with 1.5 uL of Polybrene (Santa Cruz Biotech, TX, USA). After 2 days, cells were harvested and analysed by flow cytometry.

In vivo bioluminescence imaging and acquisition of in vivo bioluminescence spectra.
'Animal work was carried out in accordance with Home Office regulations and guidelines as set out by the 1986 Animals Act (Scientific Procedures). Prior to imaging, mice were anaesthetised using 4% isoflurane and 2 L/min oxygen and during imaging were maintained

using using 1.5% isoflurane and 1 L/ min oxygen and prior to imaging mice were ip injected with 2 mg of substrates. Mice were placed on a 37 ^{o}C heated bed in the Photon Imager Optima (Biospace Labs, Paris, France) and imaged for 20 min – 40 min. In vivo bioluminescence spectra were collected by sequential 15 s, 30 s or 1 min acquisitions with the open filter, different filters and then open filter again. Spectra were corrected for signal decay during acquisition. Images were processed using M3 Vision software (Biospace Labs, Paris, France). Firefly luciferin potassium salt was purchased from Regis Technologies (IL, USA).

Figure 3: A mouse model of colon carcinoma was prepared by subcutaneous injection of 5 x 10^6 LS174T cells into the flanks of nude mice (CD1/ NuNu) expressing WT Fluc and tumours were allowed to develop for 4 days prior to imaging and imaged with 2 mg **1** Et ester or **6** Me ester with a 30 s integration time. Models of lymphoma were prepared by intravenous injection of 1 x 10^6 Fluc expressing Raji cells into NOD scid gamma (NSG) mice and imaging one week post inoculation for 15 s or 1 min with 2 mg **1** Et ester or **6** Me. In an orthotopic model, liver metastases were induced in nude mice (MF1/ NuNu) by intrasplenic implantation of 2 x10^6 SW1222 cells (colon carcinoma cell line) expressing Fluc and subsequent splenectomy. Mice were imaged three weeks later, and were imaged 15 min after ip injection of 2 mg **1** or 4 mg enantioimpure (40% ee) **6** Me ester for 15 s.

Figure 4: iCCD in vitro transmission of x5 Fluc with substrates in the presence of whole blood. 100µl of 10mg/ml **6** Me ester was diluted in 500µl TEM buffer and 90U of PLE was added and incubated for 2 h at 37°C. To initiate assays, 20 µl of 1.52 mg/ml D-**1** potassium salt or the product the above reaction was added to 10µl ATP and 10µl 1 mg/ml pure x5 enzyme with either 135µl PBS or whole human blood. Light emission was captured after 30 s in the Photon Imager (Biospace Labs, Paris, France).

Supplementary Figure S1.a. pH dependence of fluorescence spectra of native 1.

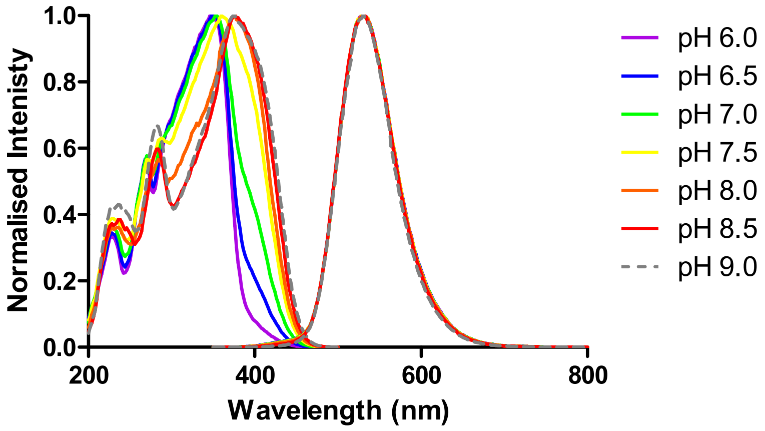

Supplementary Figure S1.b. pH dependence of fluorescence spectra of 6.

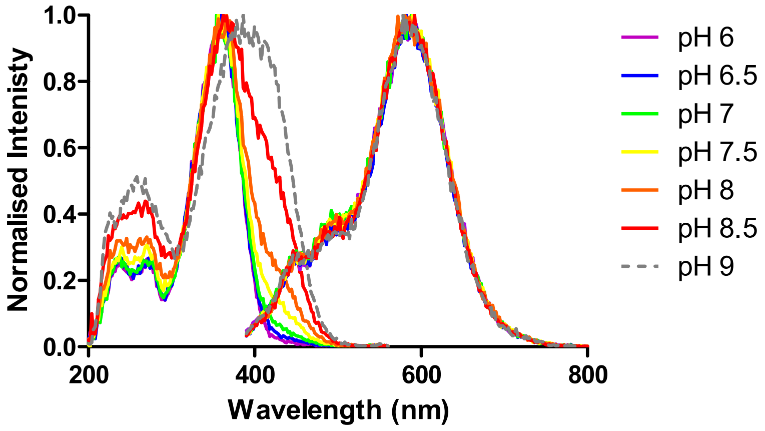

Supplementary Figure S1.c. pH dependence of fluorescence spectra of the Maki red analogue (5).

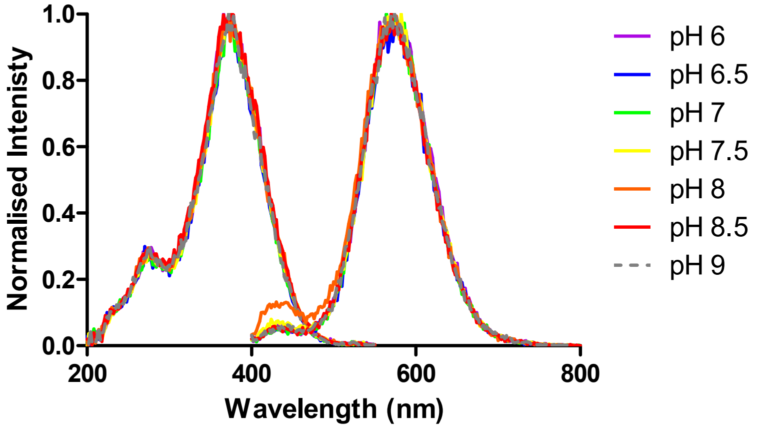

Supplementary Figure S2.a. Bioluminescence spectra of x5 Fluc and x5 S284T with 5.

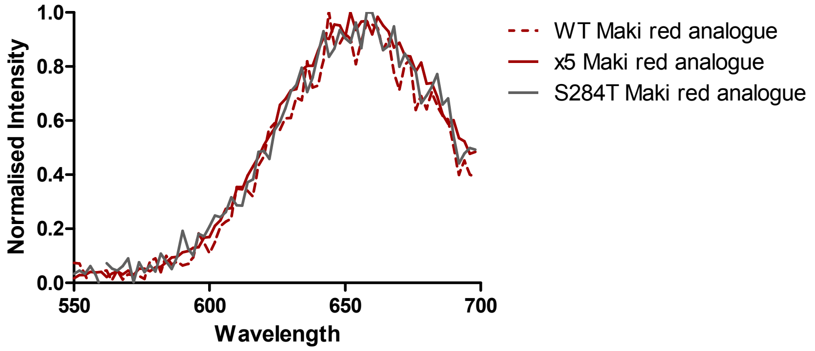

Supplementary Figure S2.b. iCCD bioluminescence spectra of pure x5 Fluc and x5 S284T with LH_2 (1) and iLH_2 (6).

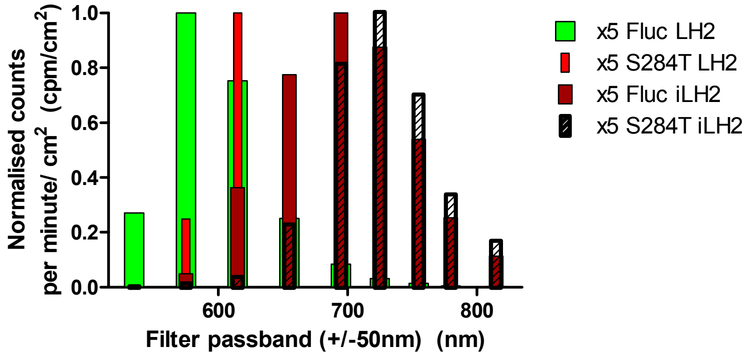

Bioluminescence spectra of native 1 (LH_2) and 6 (iLH_2). Bioluminescence spectra were acquired in a Photon Imager (Biospace Labs, Paris, France) which contains a sensitive intensified charge-coupled device (iCCD) detector with good red spectral sensitivity, by multiple consecutive 30s acquisitions using different band pass filters and correcting each output for overall signal decay. This enabled the determination of the proportion of light emitted at each wavelength for **1** (**LH_2**) and **6** (**iLH_2**) with different mutants.

Supplementary Figure S3. In vivo bioluminescence spectra of WT Fluc with LH_2 (1) and iLH_2 (6) esters in a subcutaneous model of colon carcinoma.

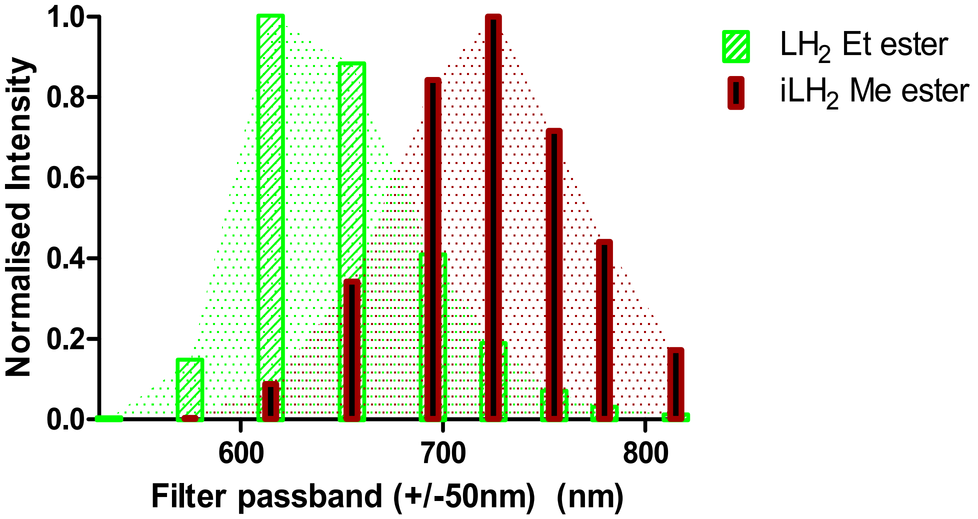

In vivo bioluminescence spectra of WT Fluc with iLH_2 methyl ester and LH_2 ethyl ester in a subcutaneous colon carcinoma (LS174T) model. 2 mg of LH_2 ethyl ester in phosphate buffered saline or 2 mg iLH_2 methyl ester in dimethyl sulfoxide was administered ip to mice and they were imaged for over time in the Photon Imager instrument (Biospace Labs, Paris, France). Light emission was captured with no filter for 30 s, then using different 50 nm pass-band filters for 30 s, followed by the open filter again in the Photon Imager.

Supplementary Figure S4. In vivo bioluminescence spectra of x5 Fluc with LH_2 (1) and iLH_2 (6) ester in a systemic lymphoma model.

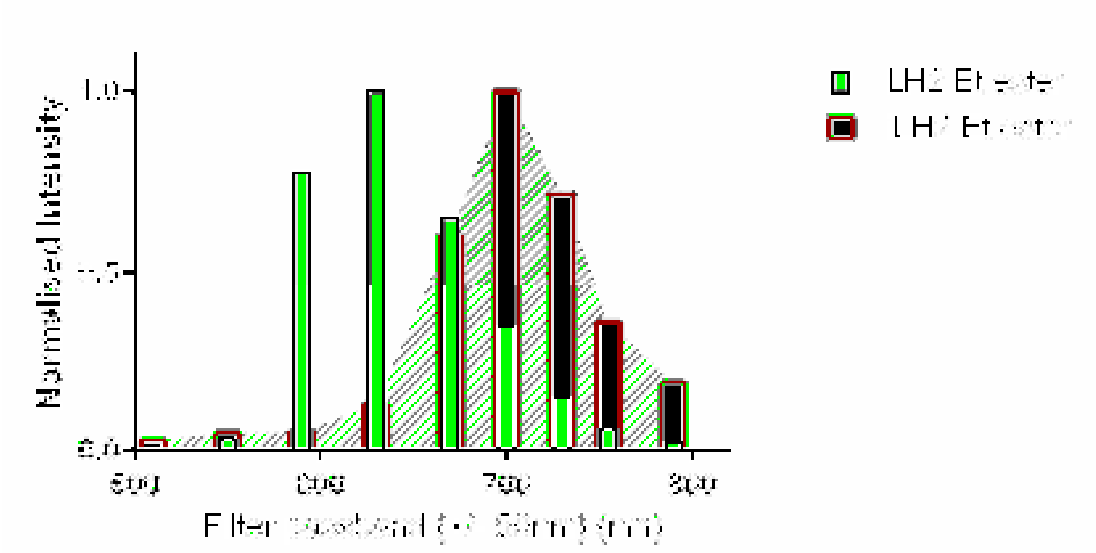

NSG mice with 1 week old systemic lymphoma expressing x5 Fluc given 2mg of substrates intraperitoneally (ip). Mice were imaged with an open filter and then sequentially through 9

bp filters with 50nm pass bands (mid-wavelength indicated in white) before imaging through the open filter. Mice were imaged for 30s or 1min with LH $_2$and iLH$_2$, respectively.

Supplementary Figure S5. In vivo bioluminescence spectra of WT Fluc with LH$_2$ (1) and iLH$_2$ (6) ester in orthotopic models of liver mestastases.

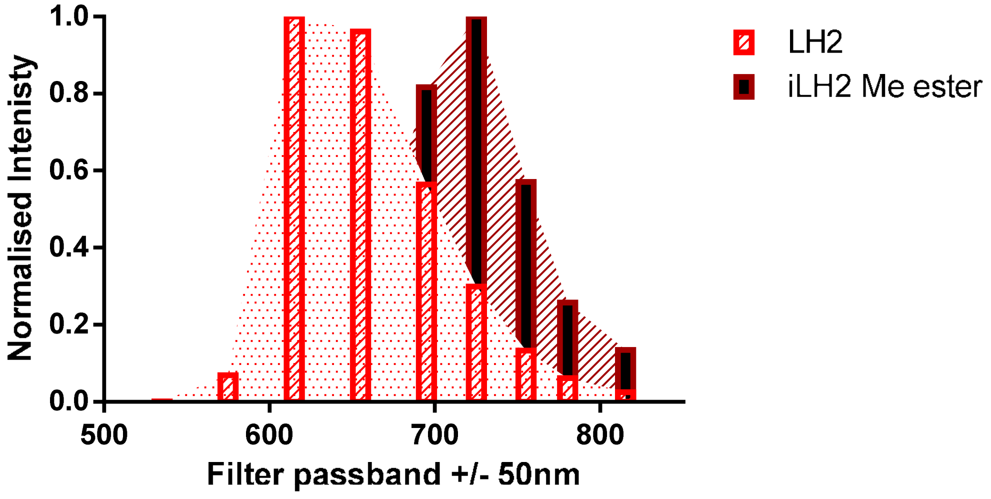

See Figure 4 legend (C) for details. Acquisition of spectra as in S4, but 15 s integration times used for both substrates.

General Experimental Details.

All manipulations were routinely carried out under an inert (Ar or N_2) atmosphere. All reagents were used as received unless stated. For the purposes of thin layer chromatography (tlc), Merck silica-aluminium plates were used, with *uv* light (254 nm) and potassium permanganate or anisaldehyde for visualisation. For column chromatography Merck Geduran® Si 60 silica gel was used. Butyl lithium solutions were standardised with diphenyl acetic acid.

All NMR data was collected using a Bruker AMX 300 MHz, Bruker AVANCE 500 MHz or Bruker AVANCE III 600 MHz. Reference values for residual solvents were taken as δ = 7.27 ($CDCl_3$), 2.51 (DMSO –*d*6), 3.30 (MeOD- *d*4) ppm for ^{1}H NMR and δ = 77.2 ($CDCl_3$), 39.5 (DMSO –*d*6), 49.0 (MeOD- *d*4) ppm for ^{13}C NMR. Coupling constants (*J*) are given in Hz and are uncorrected. Where appropriate COSY and DEPT experiments were carried out to aid assignment. Mass spectroscopy data was collected on a Thermo Finnigan Mat900xp (EI/CI) and Waters LCT Premier XE (ES) instruments. Elemental analysis was performed on an Exeter Analytical Inc. EA440 horizontal load analyser. Melting points are uncorrected and were recorded on a Stuart Scientific SMP3 syatem. Optical rotations were obtained using a Perkin-Elmer 343 digital polarimeter and are reported in deg cm^2 g^{-1}. Chiral HPLC was performed using either a Chiralcel AD 25 cm analytical column or OD-H 15 cm analytical column.

6-Hydroxy-2-bromo-benzothiazole (**S1**) was synthesised in two steps from commercially available 6-methoxy-2-amino-benzothiazole using literature procedures.[4,5] Amino acid, D-TrtCysOMe[6] was synthesised using an analogous method to that developed for L-cysteine by Rudolph *et al.*, and protected as the methyl ester using thionyl chloride.[7]

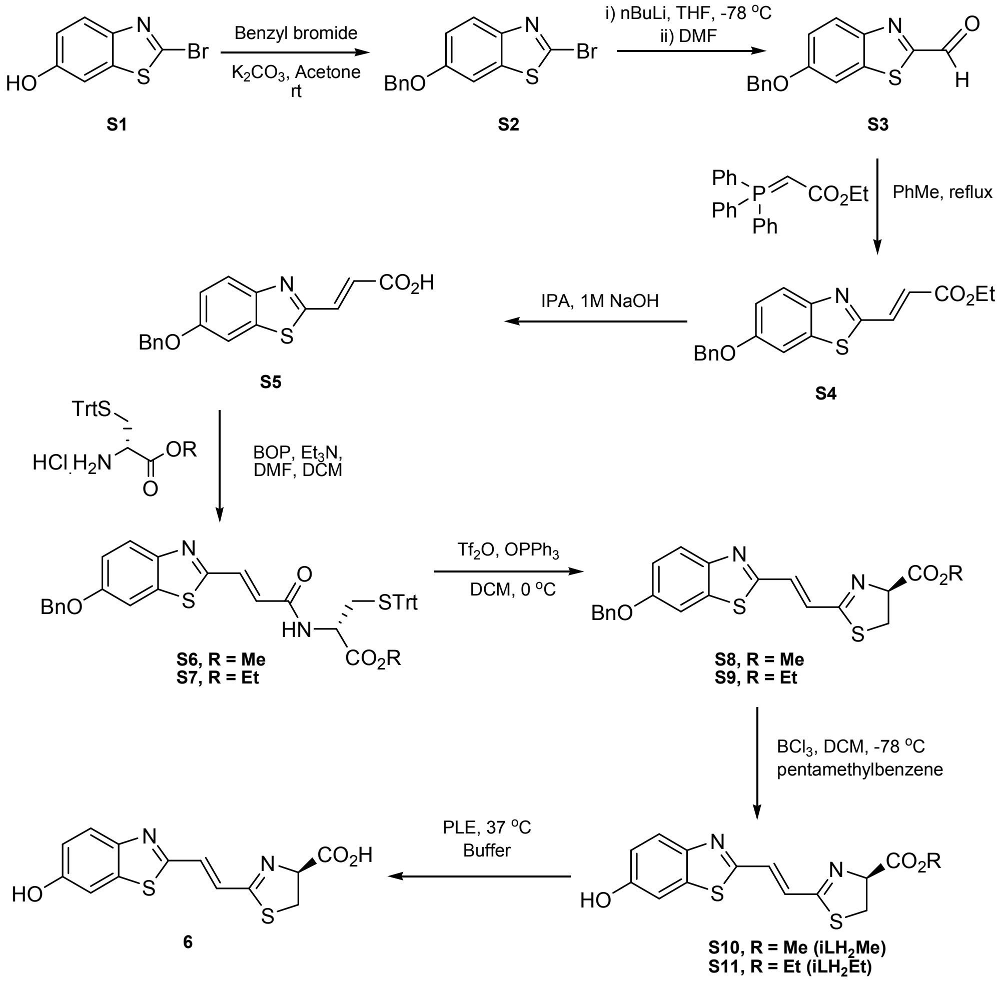

6-Benzoxy-2-bromo-benzothiazole (**S2**)

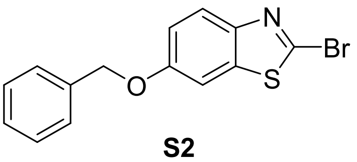

S2

A solution of **S1** (640 mg, 2.78 mmol) and BnBr (0.38 mL, 3.33 mmol) in acetone (50 mL) was treated with K_2CO_3 (1.09 g, 7.77 mmol) and stirred at rt for 16 h. After this time the reaction was filtered and concentrated *in vacuo*. Purification was achieved by flash column chromatography (5 % EtOAc/Pet. Ether) to give **S2** (759 mg, 85 %) as a white solid, m.p. 58-60 °C; R_f = 0.61 (20 % EtOAc/Pet. Ether); IR ν_{max} 2937 (ν_{CH}), 2873 (ν_{CH}), 1598, 1557, 1483, 1452, 1380, 1282, 1246, 1222, 1210, 1016, 946 cm^{-1}; ^1H NMR (500 Hz, $CDCl_3$) δ 5.13 (2H, s, OCH_2Ar), 7.14 (1H, dd, *J* = 9.0, 2.6, Ar*H*), 7.33 (1H, d, *J* = 2.5, Ar*H*), 7.35-7.46 (6H, m, Ar*H*), 7.87 (1H, d, *J* = 8.9, Ar*H*); ^{13}C NMR (125 Hz, $CDCl_3$) δ 70.8 (CH_2), 105.0 (CH), 116.5 (CH), 123.4 (CH), 127.5 (CH), 128.3 (CH), 128.8 (CH), 135.7 (C), 136.4 (C), 138.6 (C), 147.1 (C), 157.2 (C); m/z (ES+) 322 (100 %), 320 (100 %, M^++H), 321 (18 %), 272 (7 %),

272 (7 %); HRMS $C_{14}H_{11}BrNOS$ calcd. 319.9745, found 319.9730; Anal. Calcd. for $C_{14}H_{10}BrNOS$: C, 52.51; H, 3.15; N, 4.37. Found C, 52.19; H, 3.03; N, 4.33 %.

6-Benzoxy-2-formyl-benzothiazole (**S3**)

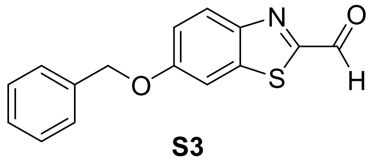

S3

A solution of **S2** (120 mg, 0.374 mmol) in THF (4 mL) was cooled to -78 °C, treated with ^{n}BuLi (1.93 M, 0.22 mL, 0.412 mmol) (colour change from colourless to pale yellow) and stirred at -78 °C for 15 min. After this time DMF (0.12 mL, 1.55 mmol) was added dropwise and the resultant solution stirred at -78 °C for 1 h. The reaction was quenched with $NaHCO_3$ $_{(aq)}$ (2 mL), back extracted with EtOAc (2 × 10 mL), separated and concentrated *in vacuo*. Purification was achieved by flash column chromatography (5-10 % EtOAc/Pet. Ether) to give **S3** (98 mg, 96 %) as a white solid, m.p. 112-114 °C; R_f = 0.51 (20 % EtOAc/Pet. Ether); IR ν_{max} 3035 (ν_{CH}), 2854 (ν_{CH}), 1677 (ν_{CO}), 1598, 1547, 1489, 1455, 1380, 1269, 1192, 1120, 999 cm^{-1}; ^{1}H NMR (500 Hz, $CDCl_3$) δ 5.19 (2H, s, OCH_2Ar), 7.30 (1H, dd, *J* = 9.1, 2.5, Ar*H*), 7.38-7.48 (7H, m, Ar*H*), 8.12 (1H, d, *J* = 9.1, Ar*H*), 10.1 (1H, s, C(O)*H*); ^{13}C NMR (125 Hz, $CDCl_3$) δ 70.8 (CH_2), 105.0 (CH), 118.8 (CH), 126.7 (CH), 127.6 (CH), 128.5 (CH), 128.9 (CH), 136.0 (C), 138.6 (C), 148.5 (C), 159.5 (C), 163.2 (C), 185.2 (CH); m/z (ES+) 270 (100 %, M^++H), 210 (6 %), 132 (14 %, M^++H-C(O)H, OBn); HRMS $C_{15}H_{12}NO_2S$ calcd. 270.0589, found 270.0585; Anal. Calcd. for $C_{15}H_{11}NO_2S$: C, 66.89; H, 4.12; N, 5.20. Found C, 66.62; H, 4.04; N, 5.10 %.

(E)-3-(6-benzoxy-benzothiazol-2-yl)-acrylic acid ethyl ester (**S4**)

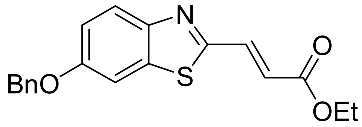

S4

A solution of **S3** (568 mg, 2.10 mmol) in PhMe (10 mL) was treated with (carbethoxymethylene)triphenylphosphorane (2.16 g, 6.31 mmol) and heated to reflux for 3 h. After this time the reaction was cooled to rt and concentrated *in vacuo*. Purification was achieved by flash column chromatography (10 % EtOAc/Pet. Ether) to give **S4** (660 mg, 92 %) as a pale yellow solid. m.p 79-80 °C; R_f = 0.38 (20 % EtOAc/Pet. Ether); IR ν_{max} 3063 (ν_{CH}), 3038 (ν_{CH}), 2979 (ν_{CH}), 1703 (ν_{CO}) 1595, 1551, 1488, 1445, 1386, 1368, 1254, 1106, 1018, 958 cm^{-1}; ^{1}H NMR (500 Hz, $CDCl_3$) δ 1.36 (3H, t, *J* = 7.1, CH_2CH_3), 4.31 (2H, q, *J* = 7.1, C$H_2$$CH_3$), 5.16 (2H, s, OC$H_2$Ar), 6.71 (1H, d, *J* = 15.9, C*H*C(O)OEt), 7.20 (1H, dd, *J* =

9.0, 2.5, Ar*H*), 7.35-7.48 (6H, m, Ar*H*), 7.85 (1H, d, *J* = 15.9, C*H*C(N)S), 7.96 (1H, d, *J* = 9.0, Ar*H*); ^{13}C NMR (125 Hz, $CDCl_3$) δ 14.3 (CH_3), 61.2 (CH_2), 70.7 (CH_2), 105.2 (CH), 117.3 (CH), 124.7 (CH), 125.1 (CH), 127.6 (CH), 128.3 (CH), 128.8 (CH), 136.4 (C), 136.8 (C), 137.0 (CH), 148.7 (C), 158.0 (C), 161.2 (C), 165.8 (C); m/z (ES+) 340 (100%, M^++H), 339 (7%, M^+), 294 (7%, M^++H-OEt), 91 (7%, $PhCH_2$); HRMS $C_{19}H_{18}NO_3S$ calcd. 340.1007, found 340.1003; Anal. Calcd. for $C_{19}H_{17}NO_3S$: C, 67.24; H, 5.05; N, 4.13. Found C, 67.04; H, 5.02; N, 4.09 %.

(E)-3-(6-benzoxy-benzothiazol-2-yl)-acrylic acid (**S5**)

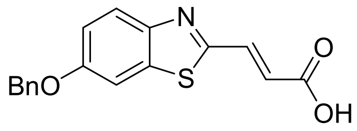

S5

A suspension of **S4** (500 mg, 1.47 mmol) in ^{i}PrOH (18.3 mL) was treated with NaOH (4.70 mL, 1.0 M) and stirred at rt overnight. An additional 10 mL of H_2O was added so that all precipitate had dissolved and the resultant solution acidified to pH 1 with HCl (1 M). The yellow precipitate was isolated by filtration and washed with H_2O (30 mL) to give **S5** (458 mg, quant.) as a fine yellow powder. m.p 174-176 °C; R_f = 0.40 (10 % MeOH/DCM); IR ν_{max} 3031 (ν_{CH}), 2866 (ν_{CH}), 2543 (ν_{OH}), 1694 (ν_{CO}), 1599, 1558, 1497, 1451, 1312, 1259, 1205, 1051, 1013, 966 cm^{-1}; ^{1}H NMR (300 Hz, DMSO) δ 5.20 (2H, s, OC*H*$_2$Ar), 6.72 (1H, d, *J* = 15.9, C*H*C(O)OEt), 7.23 (1H, dd, *J* = 9.0, 2.5, Ar*H*), 7.32-7.50 (5H, m, Ar*H*), 7.69 (1H, d, *J* = 15.9, C*H*C(N)S), 7.83 (1H, d, *J* = 2.4, Ar*H*), 7.97 (1H, d, *J* = 9.0, Ar*H*); ^{13}C NMR (125 Hz, DMSO) δ 69.9 (CH_2), 105.8 (CH), 117.2 (CH), 124.2 (CH), 126.0 (CH), 127.9 (CH), 128.0 (CH), 128.5 (CH), 135.9 (CH), 136.6 (C), 136.6 (C), 147.9 (C), 157.4 (C), 160.8 (C), 166.4 (C); m/z (CI) 402 (26%), 312 (100%, M^++H), 294 (11%, M^++H-OH), 91 (5%, $PhCH_2$); HRMS $C_{17}H_{14}NO_3S$ calcd. 312.0694, found 312.0691.

D-(Methyl 2-((E)-3-(6-benzoxybenzothiazol-2-yl)acrylamido)-3-(tritylthio)proponoate (**S6**)

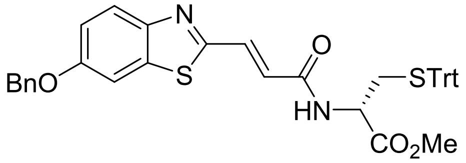

S6

A solution of **S5** (134 mg, 0.424 mmol) in DMF (4.2 mL) was treated with Et_3N (70 µL, 1.02 mmol) and cooled to 0 ºC. The solution was treated with a solution of aminoacid (192 mg, 0.508 mmol) in DCM (2 mL) followed by a solution of BOP (231 mg, 0.508 mmol) in DCM (2.2 mL) and the resultant solution stirred at 0 ºC for 2 h. After this time the reaction mixture was quenched with saturated $NaHCO_{3(aq)}$ (2 mL) and taken up in EtOAc (10 mL), the aqueous

layer was back extracted using EtOAc (2 × 5 mL), organics dried over $MgSO_4$, filtered and concentrated *in vacuo*. Purification was achieved by flash column chromatography (20 % EtOAc/Pet. Ether) to give **S6** (220 mg, 80 %) as a yellow solid. m.p. 83-86 °C; R_f = 0.28 (30 % EtOAc/Pet. Ether); $[\alpha]_D$ +62.6 (c 0.95, $CHCl_3$); CSP HPLC analysis (Chiracel OD-H (150 × 4.6 mm), eluent: hexane: ^{i}PrOH, 70:30, flow 0.5 mL/min, 14 bar) determined > 98 % ee [t_R (minor) = 43.07 min, t_R (major) = 26.59 min]; IR ν_{max} 1741 (ν_{CO}), 1662, 1627, 1596, 1488, 1446, 1346, 1319, 1260, 1196, 1051, 1001, 966 cm^{-1}; ^{1}H NMR (500 Hz, $CDCl_3$) δ 2.73 (1H, dd, *J* = 12.7, 4.6, CH_2STrt), 2.82 (1H, dd, *J* = 12.7, 5.6, CH_2STrt), 3.76 (3H, s, OCH_3), 4.76 (1H, dt, *J* = 7.8, 5.2, C*H*CH_2), 5.17 (2H, s, OCH_2Ph), 6.09 (1H, d, *J* = 7.8, N*H*), 6.65 (1H, d, *J* = 15.5, C*H*C(O)N), 7.20-7.49 (22H, m, Ar*H*), 7.73 (1H, d, *J* = 15.5, C*H*C(N)S), 7.96 (1H, d, *J* = 9.0, Ar*H*); ^{13}C NMR (150 Hz, $CDCl_3$) δ 33.8 (CH_2), 51.4 (CH), 52.9 (CH_3), 67.2 (C), 70.7 (CH_2), 105.2 (CH), 117.2 (CH), 124.6 (CH), 126.8 (CH), 127.1 (CH), 127.6 (CH), 128.1 (CH), 128.3 (CH), 128.8 (CH), 129.6 (CH), 134.2 (CH), 136.4 (C), 144.3 (C), 154.2 (C), 157.9 (C), 161.4 (C), 163.8 (C), 170.7 (C); m/z (ES+) 693 (96%, M^++Na), 595 (9%), 244 (18%), 243 (100%, M^+- STrt,CO_2Me, Bn, H), 228 (8%), 165 (22%); HRMS $C_{40}H_{34}N_2O_4NaS_2$ calcd. 693.1858, found 693.1855.

D-(Ethyl 2-((E)-3-(6-benzoxybenzothiazol-2-yl)acrylamido)- 3-(tritylthio)proponoate (**S7**)

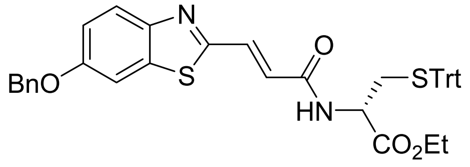

S7

As **S6**, using **S5** (239 mg, 0.755 mmol), ethylester aminoacid (354 mg, 0.908 mmol). Purification was achieved by flash column chromatography (20 % EtOAc/Pet. Ether) to give **S7** (422 mg, 82 %) as an orange solid. m.p. 75-78 °C; R_f = 0.23 (30 % EtOAc/Pet. Ether); $[\alpha]_D$ +82.0 (c 1.05, $CHCl_3$); CSP HPLC analysis (Chiracel AD (300 × 4.6 mm), eluent: hexane: ^{i}PrOH, 60:40, flow 1 mL/min, 26 bar) determined > 99 % ee [t_R (minor) = 16.52 min, t_R (major) = 9.67 min]; IR ν_{max} 1735 (ν_{CO}), 1667, 1628, 1596, 1488, 1446, 1371, 1344, 1260, 1189, 1050, 1019, 966 cm^{-1}; ^{1}H NMR (600 Hz, $CDCl_3$) δ 1.27 (3H, t, *J* = 7.2, CH_2CH_3), 2.70 (1H, dd, *J* = 12.6, 4.5, CH_2STrt), 2.80 (1H, dd, *J* = 12.6, 5.5, CH_2STrt), 4.22 (2H, m, C$H_2$$CH_3$), 4.75 (1H, dt, *J* = 7.7, 4.8, C*H*CH_2), 5.17 (2H, s, OCH_2Ph), 6.14 (1H, d, *J* = 7.8, N*H*), 6.68 (1H, d, *J* = 15.5, C*H*C(O)N), 7.20-7.49 (22H, m, Ar*H*), 7.74 (1H, d, *J* = 15.5, C*H*C(N)S), 7.96 (1H, d, *J* = 9.0, Ar*H*); ^{13}C NMR (150 Hz, $CDCl_3$) δ 14.3 (CH_3), 34.0 (CH_2), 51.5 (CH), 62.2 (CH_2), 67.1 (C), 70.7 (CH_2), 105.2 (CH), 117.2 (CH), 124.6 (CH), 126.9 (CH), 127.1 (CH), 127.6 (CH), 128.2 (CH), 128.4 (CH), 128.9 (CH), 129.6 (CH), 134.2 (CH), 136.4 (C), 136.8 (C), 144.4 (C), 148.8 (C), 157.9 (C), 161.5 (C), 163.8 (C), 170.2 (C); m/z

(ES+) 707 (100%, M^++Na), 685 (5%, M^++H), 338 (27%, M^++H-STrt, CO_2Et), 243 (100%), 228 (23%), 165 (59%); HRMS $C_{41}H_{36}NaN_2O_4S_2$ calcd. 707.2014, found 707.2039; Anal. Calcd. for $C_{41}H_{36}N_2O_4S_2$: C, 71.90; H, 5.30; N, 4.09. Found C, 71.43; H, 5.30; N, 3.98 %.

6-Benzoxy-2-(2-(4*S*-methoxycarbonyl-4,5-dihydrothiazol-2-yl)ethenyl)benzothiazole (**S8**)

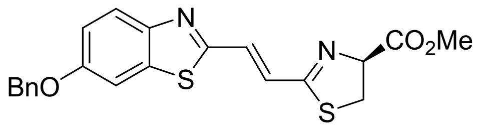

S8

A solution of Ph_3PO (386 mg, 1.40 mmol) in DCM (10 mL) was cooled to 0 °C in an ice bath and treated with Tf_2O (0.160 mL, 0.874 mmol) added dropwise over 5 min. The resultant solution was stirred at 0 °C for 30 min and a solution of **S6** (220 mg, 0.329 mmol) in DCM (4 mL) was then added dropwise over 5 min. The reaction was stirred at 0 °C for 10 min and then quenched with phosphate buffer (4 mL). Extracted into DCM (3 × 10 mL) and separated. The organic layer was dried over $MgSO_4$, filtered and concentrated *in vacuo*. Purification was achieved by flash column chromatography (20-40 % EtOAc/Pet. Ether) to give **S8** (88 mg, 65 %) as a pale yellow solid. m.p. 98-100 °C; R_f = 0.32 (40 % EtOAc/Pet. Ether); IR ν_{max} 3032, 2947 (ν_{CH}), 1726 (ν_{CO}), 1599, 1569, 1557, 1480, 1454, 1431, 1380, 1319, 1261, 1242, 1225, 1199, 1176, 1043, 1018, 946 cm^{-1}; ^1H NMR (600 Hz, $CDCl_3$) δ 3.63 (1H, dd, *J* = 10.9, 9.3, C*H*$_2$S), 3.71 (1H, dd, *J* = 11.0, 9.3, C*H*$_2$S), 3.86 (3H, s, OC*H*$_3$), 5.15 (2H, s, OC*H*$_2$Ph), 5.26 (1H, t, *J* = 9.3, C*H*CH$_2$), 7.18 (1H, dd, *J* = 9.0, 2.5, Ar*H*), 7.31-7.48 (8H, m, ArH, 2 × C*H*C(N)S), 7.93 (1H, d, *J* = 9.0, Ar*H*); ^{13}C NMR ($CDCl_3$) δ 35.1 (CH_2), 53.1 (CH_3), 70.7 (CH_2), 78.2 (CH), 105.2 (CH), 117.2 (CH), 124.6 (CH), 127.6 (CH), 128.4 (CH), 128.8 (CH), 129.0 (CH), 135.2 (CH), 136.4 (C), 136.7 (C), 148.7 (C), 158.0 (C), 162.0 (C), 169.4 (C), 170.9 (C); m/z (ES+) 474 (32%), 411 (100%, M^++H), 320 (12%); HRMS $C_{21}H_{19}N_2O_3S_2$ calcd. 411.0837, found 411.0831; Anal. Calcd. for $C_{21}H_{18}N_2O_3S_2$: C, 61.44; H, 4.42; N, 6.82. Found C, 61.38; H, 4.34; N, 6.74 %.

CSP HPLC analysis determined 45-95 % ee dependant on scale of reaction and purity of Tf_2O. Optical purity could be achieved by dissolving **S8** in MeCN and concentrating *in vacuo* until a precipitate appeared. Filtration and concentration of the filtrate gave **S8**. $[\alpha]_D$ -28.4 (c 0.91, $CHCl_3$); CSP HPLC analysis (Chiracel OD-H, eluent: hexane: ^{i}PrOH, 70:30, flow 0.5 mL/min, 14 bar) > 98 % ee [t_R (minor) = 21.8 min, t_R (major) = 30.7 min].

6-Benzoxy-2-(2-(4*S*-ethoxycarbonyl-4,5-dihydrothiazol-2-yl)ethenyl)benzothiazole **(S9)**

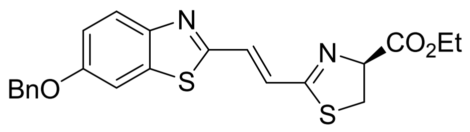

S9

As **S8**, using **S7** (544 mg, 0.797 mmol) in place of **S6**. Purification was achieved by flash column chromatography (20-40 % EtOAc/Pet. Ether) to give **S9** (250 mg, 74 %) as a pale yellow solid. m.p. 114-117 °C; $R_f = 0.39$ (40 % EtOAc/Pet. Ether); IR ν_{max} 2977 (ν_{CH}), 1729 (ν_{CO}), 1597, 1555, 1479, 1453, 1379, 1316, 1261, 1222, 1199, 1049, 1014, 947 cm^{-1}; ^1H NMR (600 Hz, $CDCl_3$) δ 1.36 (3H, t, *J* = 6.6, CH_2CH_3), 3.63 (1H, dd, *J* = 11.0, 9.4, CH_2S), 3.68 (1H, dd, *J* = 11.0, 9.4, CH_2S), 4.27-4.38 (2H, m, CH_2CH_3), 5.16 (2H, s, OCH_2Ar), 5.24 (1H, t, *J* = 9.3, $CHCO_2Et$), 7.19 (1H, dd, *J* = 9.0, 2.5, Ar*H*), 7.30-7.49 (8H, m, Ar*H*), 7.93 (1H, d, *J* = 9.0, Ar*H*); ^{13}C NMR (150 Hz, $CDCl_3$) δ 14.3 (CH_3), 35.1 (CH_2), 62.2 (CH_2), 70.7 (CH_2), 78.5 (CH), 105.2 (CH), 117.2 (CH), 124.6 (CH), 127.6 (CH), 128.4 (CH), 128.8 (CH), 129.2 (CH), 135.0 (CH), 136.4 (C), 136.6 (C), 148.7 (C), 158.0 (C), 162.1 (C), 169.2 (C), 170.5 (C); m/z (ES+) 425 (100%, M^++H), 334 (25%, M^+-Bn), 293 (11%); HRMS $C_{22}H_{21}N_2O_3S_2$ calcd. 425.0994, found 425.0998.

CSP HPLC analysis determined 82-92 % ee dependant on scale of reaction and purity of Tf_2O. Optical purity could be achieved by dissolving **S9** in MeCN and concentrating *in vacuo* until a precipitate appeared. Filtration and concentration of the filtrate gave **S9**. CSP HPLC analysis (Chiracel AD, eluent: hexane: ^{i}PrOH, 60:40, flow 1 mL/min, 26 bar) determined > 98 % ee [t_R (minor) = 23.60 min, t_R (major) = 11.19 min].

6-Hydroxy-2-(2-(4*S*-methoxycarbonyl-4,5-dihydrothiazol-2-yl)ethenyl)benzothiazole (**S10**)

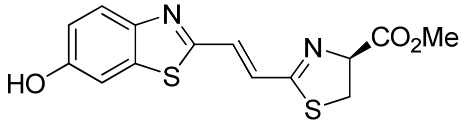

S10

A solution of **S8** (55 mg, 0.133 mmol) in DCM (2 mL) was cooled to -78 °C in an acetone/$CO_{2(s)}$ bath and treated with pentamethylbenzene (110 mg, 0.580 mmol) followed by BCl_3 (0.39 mL, 0.399 mmol, 1 M in DCM) added dropwise over 5 min. The reaction was stirred at -78 °C for 20 min and then quenched with phosphate buffer (2 mL). Extracted into DCM (3 × 5 mL) and separated. The organic layer was dried over $MgSO_4$, filtered and concentrated *in vacuo*. Purification was achieved by flash column chromatography (40-100 % EtOAc/Pet. Ether) to give **S10** (34 mg, 79 %, 98 % b.r.s.m) as a pale yellow solid. m.p. 163-165 °C; $R_f = 0.13$ (40 % EtOAc/Pet. Ether); IR ν_{max} 3374 (ν_{OH}), 2992 (ν_{CH}), 1728 (ν_{CO}) 1592,

1568, 1476, 1449, 1379, 1369, 1321, 1254, 1192, 1062, 1027, 944 cm^{-1}; ^{1}H NMR (600 Hz, MeOD) δ 3.70 (1H, dd, *J* = 20.1, 11.2), 3.72 (1H, dd, *J* = 20.8, 11.2), 3.81 (3H, s), 5.33 (1H, t, *J* = 9.0), 7.03 (1H, dd, *J* = 8.9, 2.5), 7.29 (1H, d, *J* = 16.1), 7.31 (1H, d, *J* = 2.3), 7.41 (1H, d, *J* = 16.1), 7.82 (1H, d, *J* = 8.9); ^{13}C NMR (150 Hz, MeOD) δ 35.7 (CH_2), 53.2 (CH_3), 79.0 (CH), 107.4 (CH), 118.1 (CH), 125.1 (CH), 128.9 (CH), 136.0 (CH), 138.1 (C), 148.6 (C), 158.5 (C), 162.2 (C), 171.6 (C), 172.2 (C); HRMS m/z (EI) $C_{14}H_{12}N_2O_3S_2$ calcd. 320.0284, found 320.0279.

CSP HPLC analysis determined 65-90 % ee dependant on scale of reaction and purity of BCl_3. Optical purity could be achieved by dissolving **S10** in MeCN and concentrating *in vacuo* until a precipitate appeared. Filtration and concentration of the filtrate gave **S10**. CSP HPLC analysis (Chiracel AD, eluent: hexane: ^{i}PrOH, 70:30, flow 0.5 mL/min, 23 bar) > 98 % ee [t_R (major) = 6.35 min, t_R (minor) = 9.90 min].

6-Hydroxy-2-(2-(4*S*-ethoxycarbonyl-4,5-dihydrothiazol-2-yl)ethenyl)benzothiazole (**S11**)

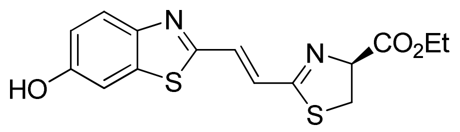

S11

As **S10**, using **S9** (67 mg, 0.158 mmol) in place of **S8**. Purification was achieved by flash column chromatography (40-100 % EtOAc/Pet. Ether) to give **S11** (38 mg, 72 %, 96 % b.r.s.m) as a pale yellow solid. m.p. 155-157 °C; R_f = 0.11 (40 % EtOAc/Pet. Ether); IR ν_{max} 3377 (ν_{OH}), 2983 (ν_{CH}), 1732 (ν_{CO}) 1592, 1568, 1476, 1449, 1379, 1369, 1321, 1281, 1254, 1192, 1116, 1062, 1027, 944 cm^{-1}; ^{1}H NMR (600 Hz, $CDCl_3$) δ 1.36 (3H, t, *J* = 7.1, CH_2CH_3), 3.64 (1H, dd, *J* = 20.5, 10.8, CH_2S), 3.68 (1H, dd, *J* = 20.3, 10.8, CH_2S), 4.30-4.36 (2H, m, CH_2CH_3), 5.25 (1H, t, *J* = 9.2, $CHCO_2Et$), 5.76 (1H, s, O*H*), 7.03 (1H, dd, *J* = 8.8, 2.4, Ar*H*), 7.28 (1H, d, *J* = 16.1, C*H*C(N)S) 7.30 (1H, d, *J* = 2.4, Ar*H*), 7.38 (1H, d, *J* = 16.1, C*H*C(N)S), 7.89 (1H, d, *J* = 8.8, Ar*H*); ^{13}C NMR (150 Hz, $CDCl_3$) δ 14.3 (CH_3), 35.1 (CH_2), 62.3 (CH_2), 78.4 (CH), 107.0 (CH), 116.7 (CH), 124.7 (CH), 129.1 (CH), 135.0 (CH), 136.7 (C), 148.6 (C), 155.0 (C), 162.0 (C), 169.5 (C), 170.6 (C); m/z (ES+) 335 (100%, M^++H), 307 (12%, M^++H-Et), 261 (48%, M^++H-CO_2Et), 203 (47 %, M^++H-SCH_2CHCO_2Et); HRMS $C_{15}H_{15}N_2O_3S_2$ calcd. 335.0524, found 335.0526.

CSP HPLC analysis determined 80-92 % ee dependant on scale of reaction and purity of BCl_3. Optical purity could be achieved by dissolving **S11** in MeCN and concentrating *in vacuo* until a precipitate appeared. Filtration and concentration of the filtrate gave **S11.** CSP

HPLC analysis (Chiracel AD, eluent: hexane: ^{i}PrOH, 70:30, flow 1.0 mL/min, 23 bar) > 98 % ee [t_R (major) = 6.85 min, t_R (minor) = 9.30 min].

6-Hydroxy-2-(2-(4*S*-carboxy-4,5-dihydrothiazol-2-yl)ethenyl)benzothiazole (**6**)

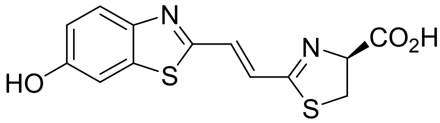

6

A solution of **S10** or **S11** in phosphate buffer (1 mg/mL) was treated with PLE (5 µg per 1 mg of ester) and incubated at 37 °C for 24 h. After this time the saponified ester **6** was used directly without isolation.

^{1}H NMR spectrum of **S2** ($CDCl_3$)

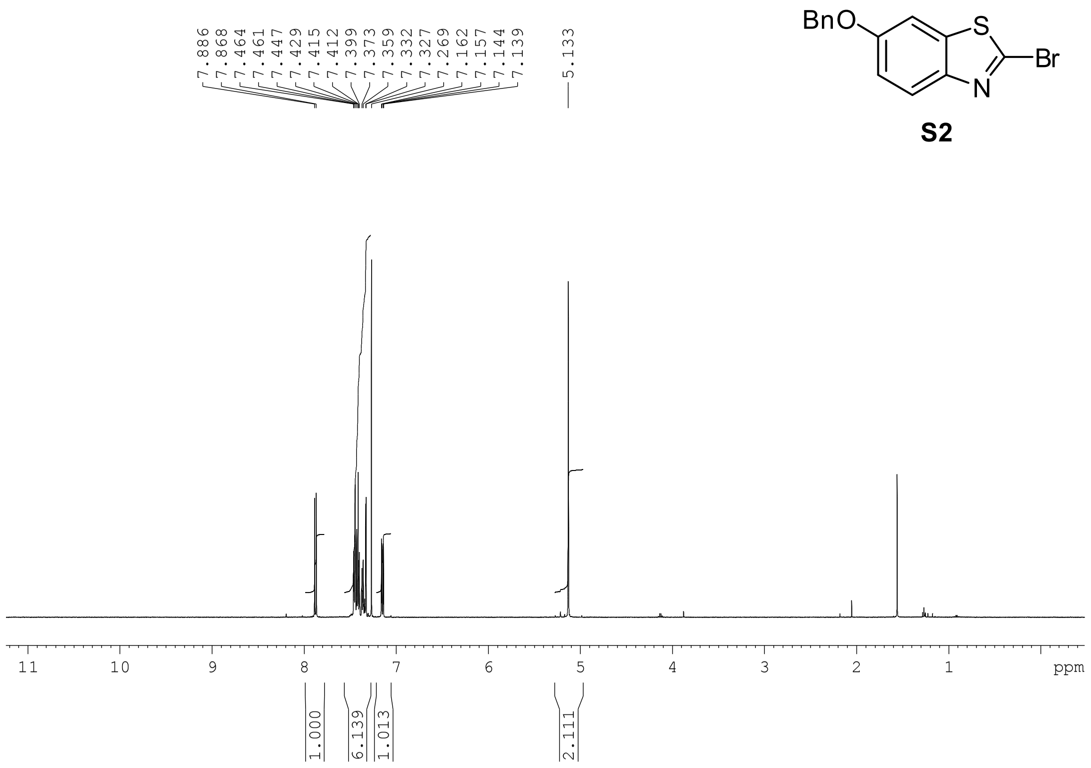

^{13}C NMR spectrum of **S2** ($CDCl_3$)

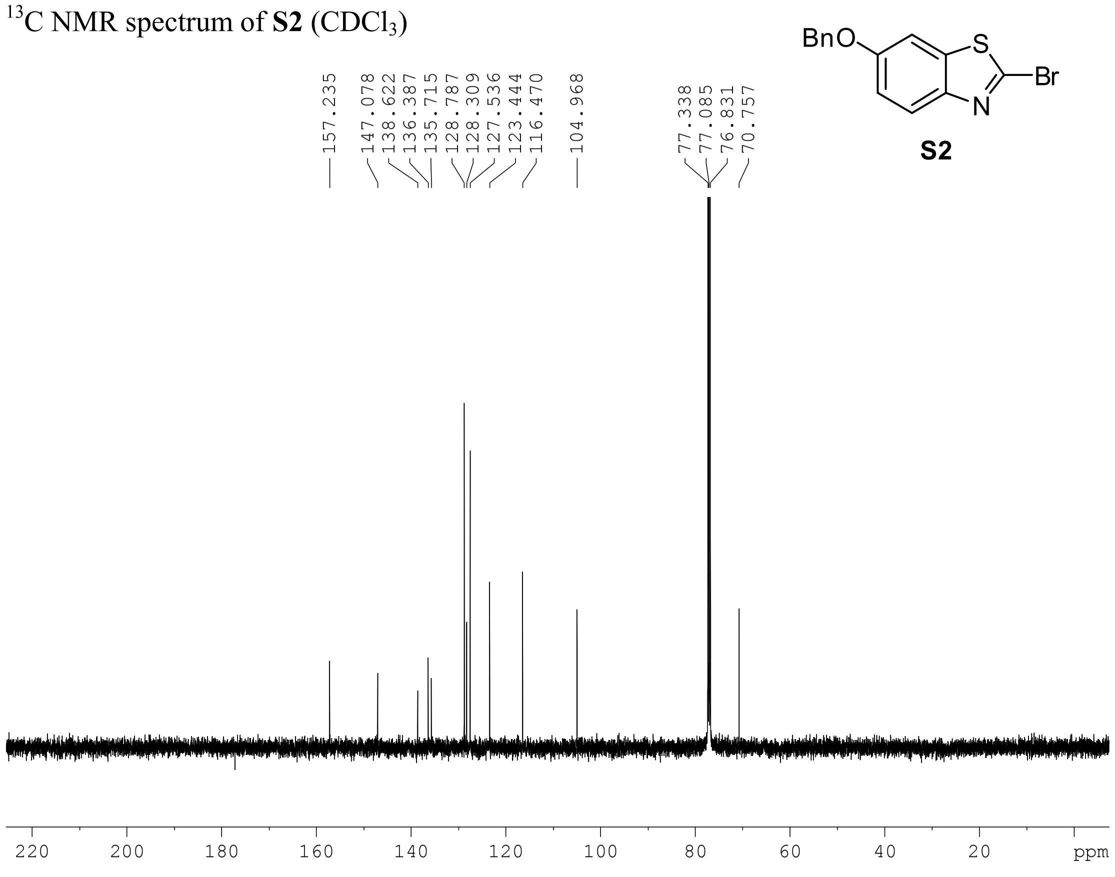

^{1}H NMR spectrum of **S3** ($CDCl_3$)

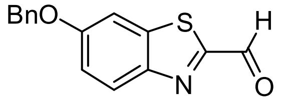

S3

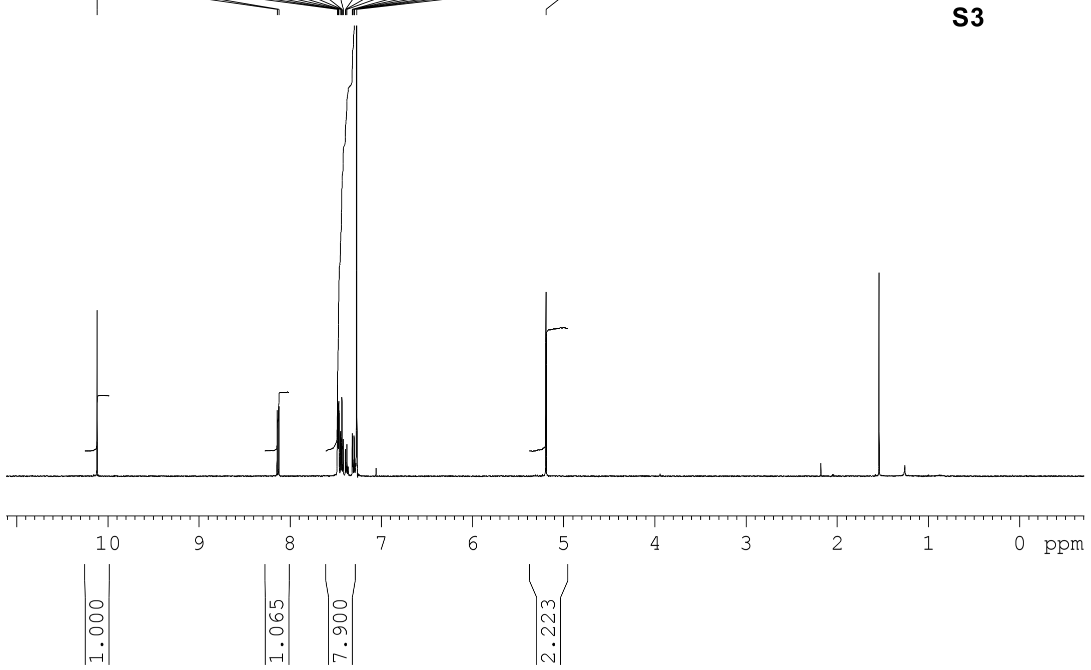

^{13}C NMR spectrum of **S3** ($CDCl_3$)

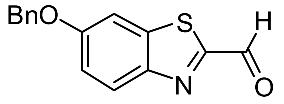

S3

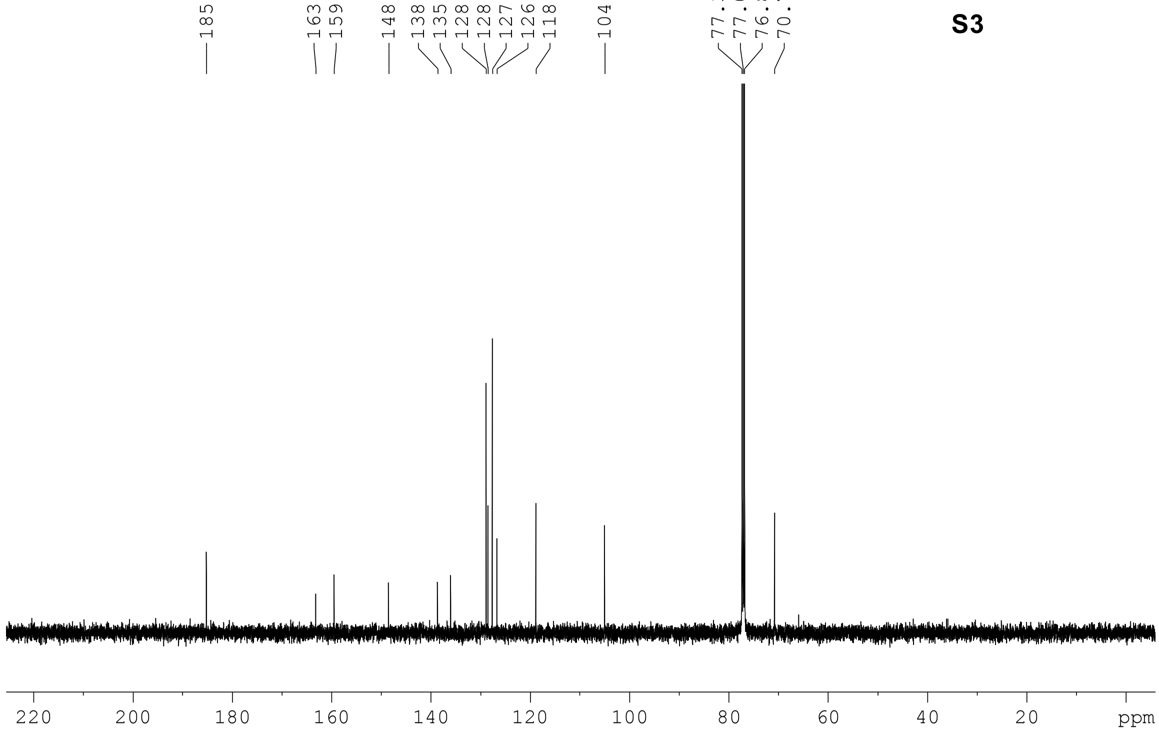

^{1}H NMR spectrum of **S4** ($CDCl_3$)

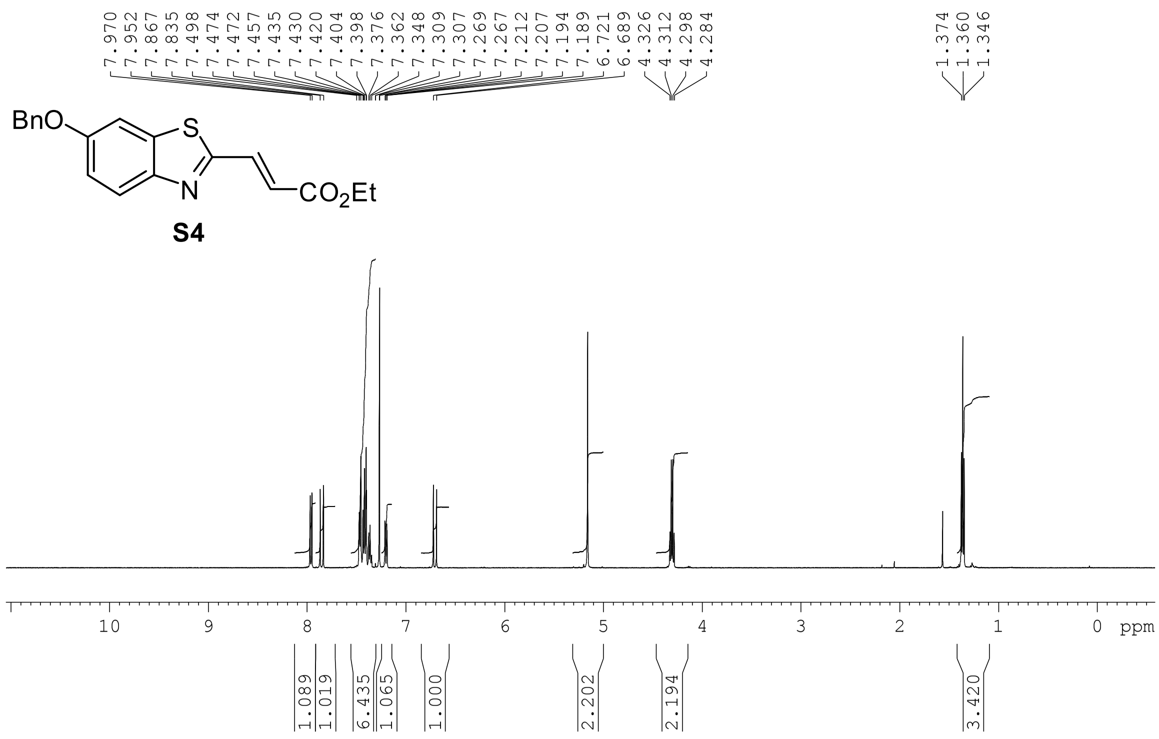

^{13}C NMR spectrum of **S4** ($CDCl_3$)

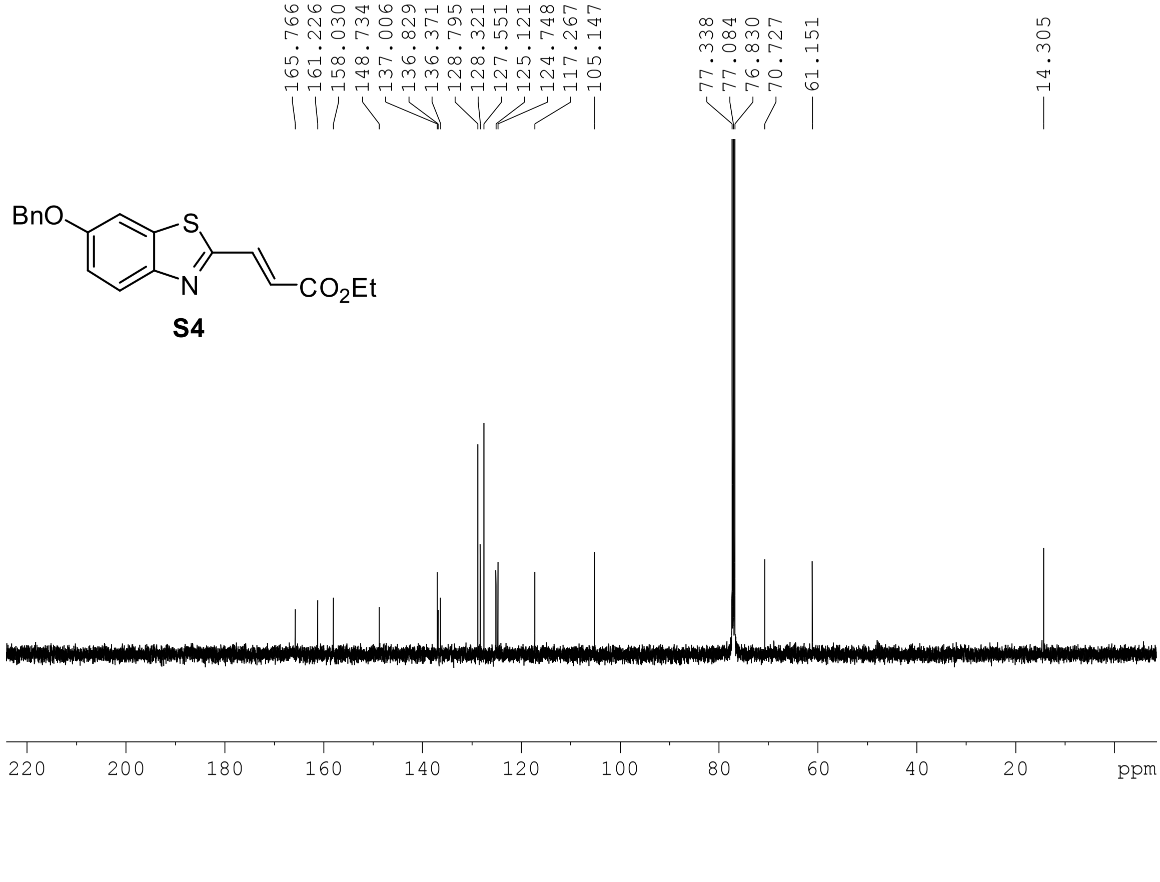

^1H NMR spectrum of **S5** (DMSO)

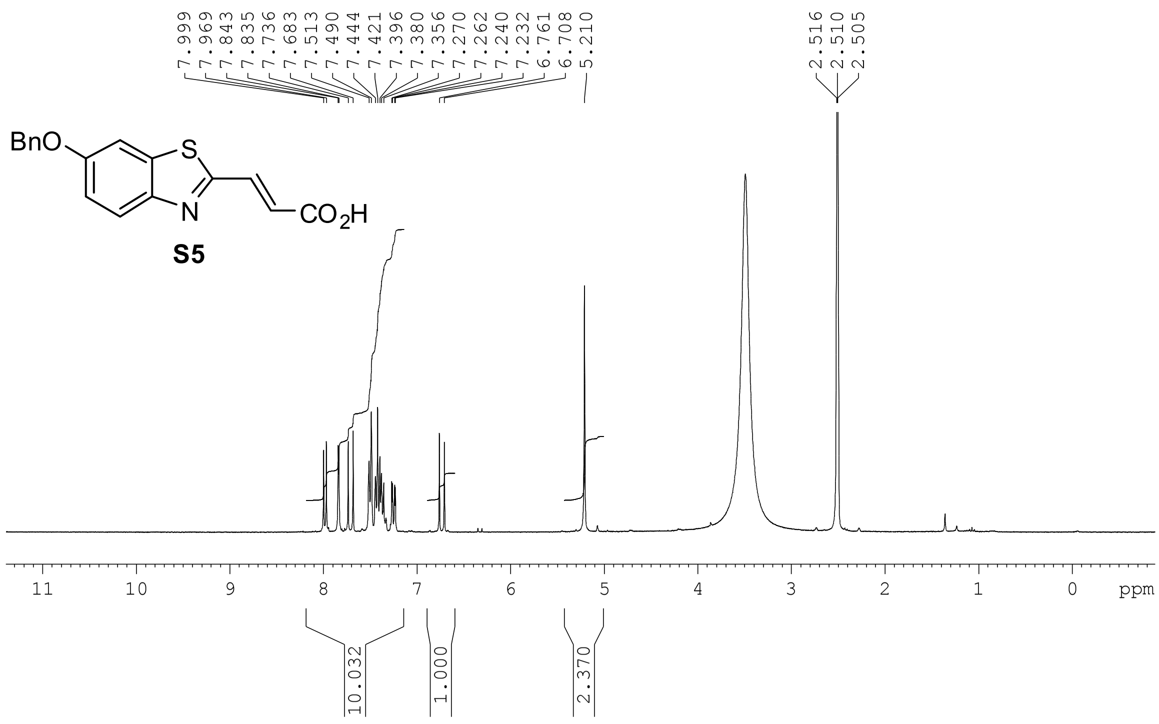

^{13}C NMR spectrum of **S5** (DMSO)

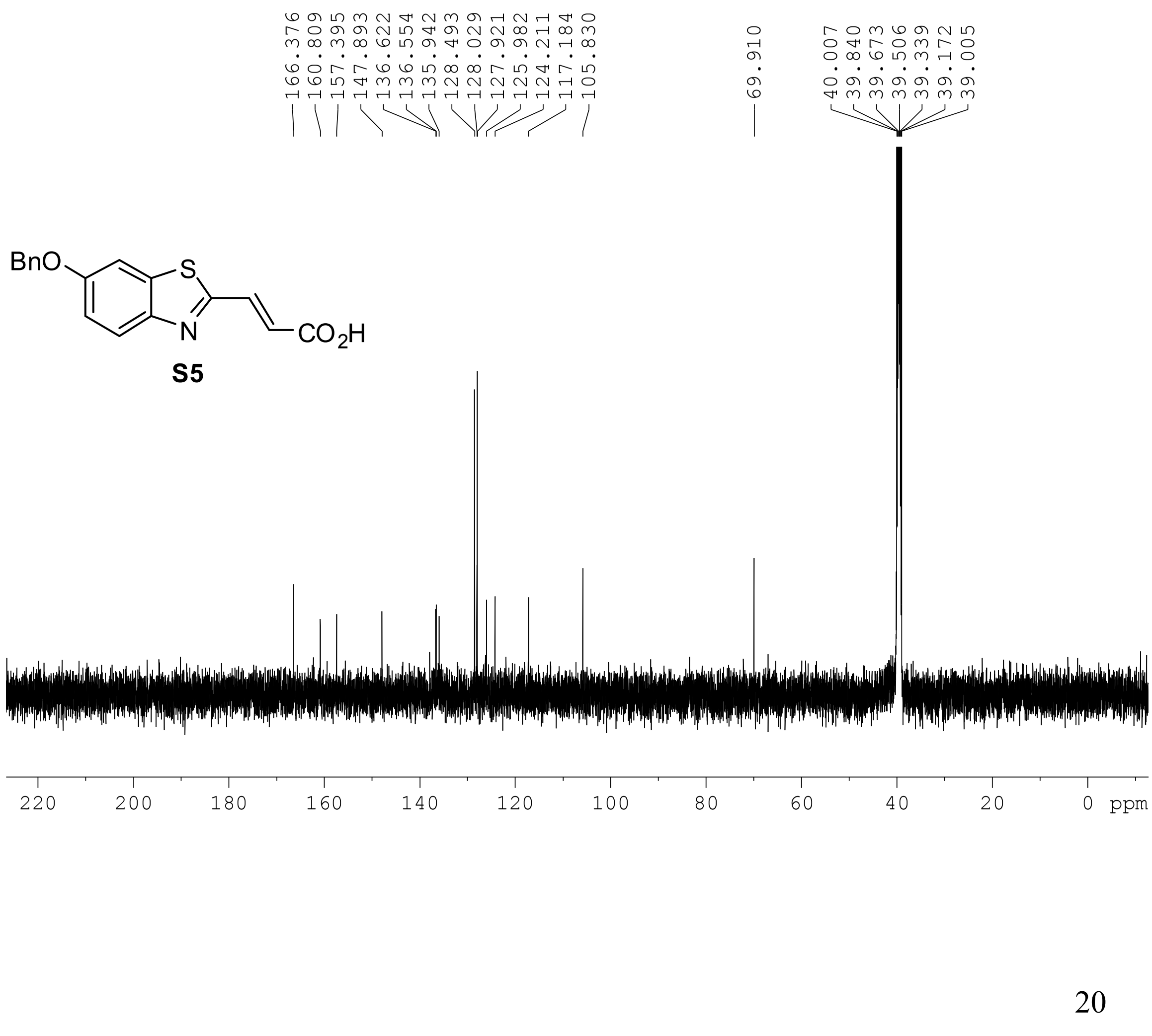

^{1}H NMR spectrum of **S6** ($CDCl_3$)

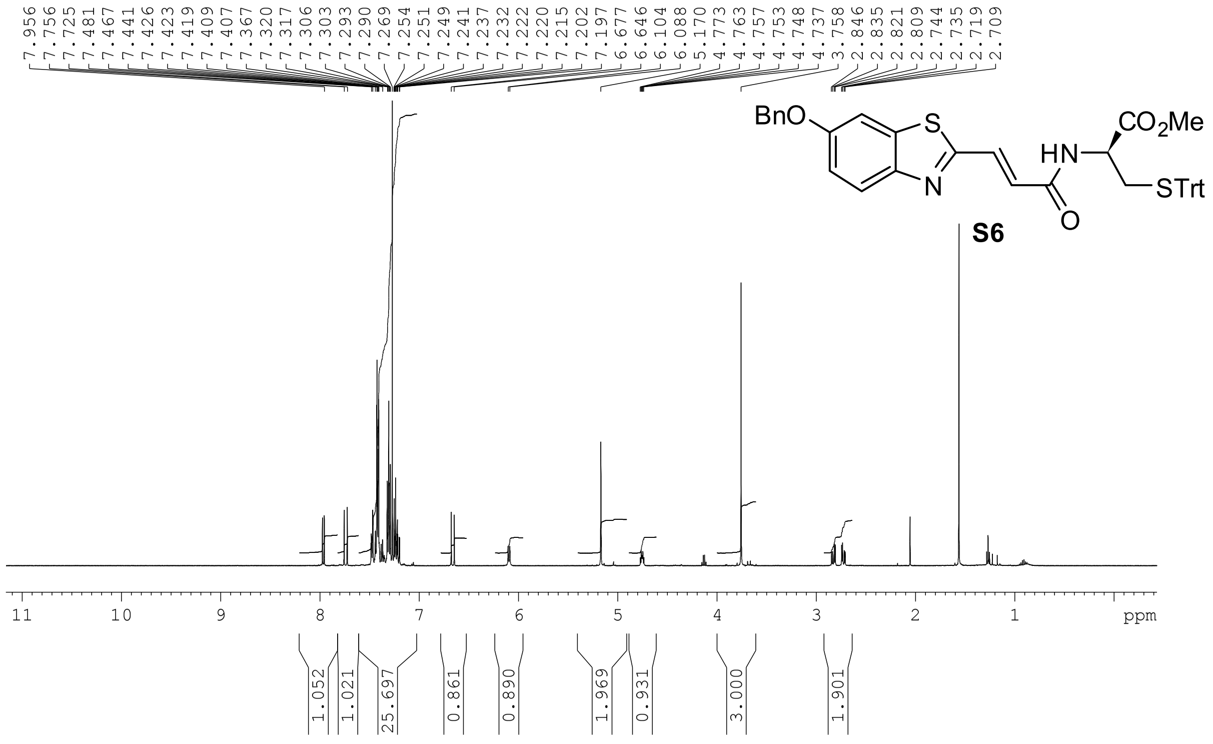

^{13}C NMR spectrum of **S6** ($CDCl_3$)

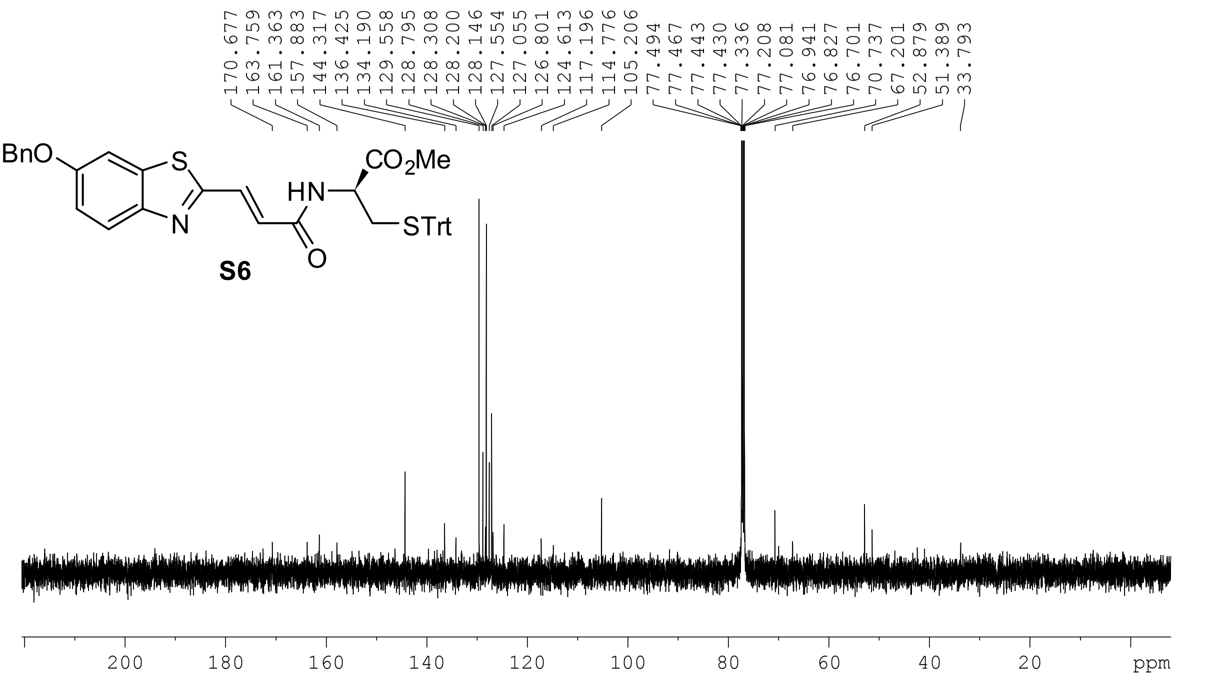

^{1}H NMR spectrum of **S7** ($CDCl_3$)

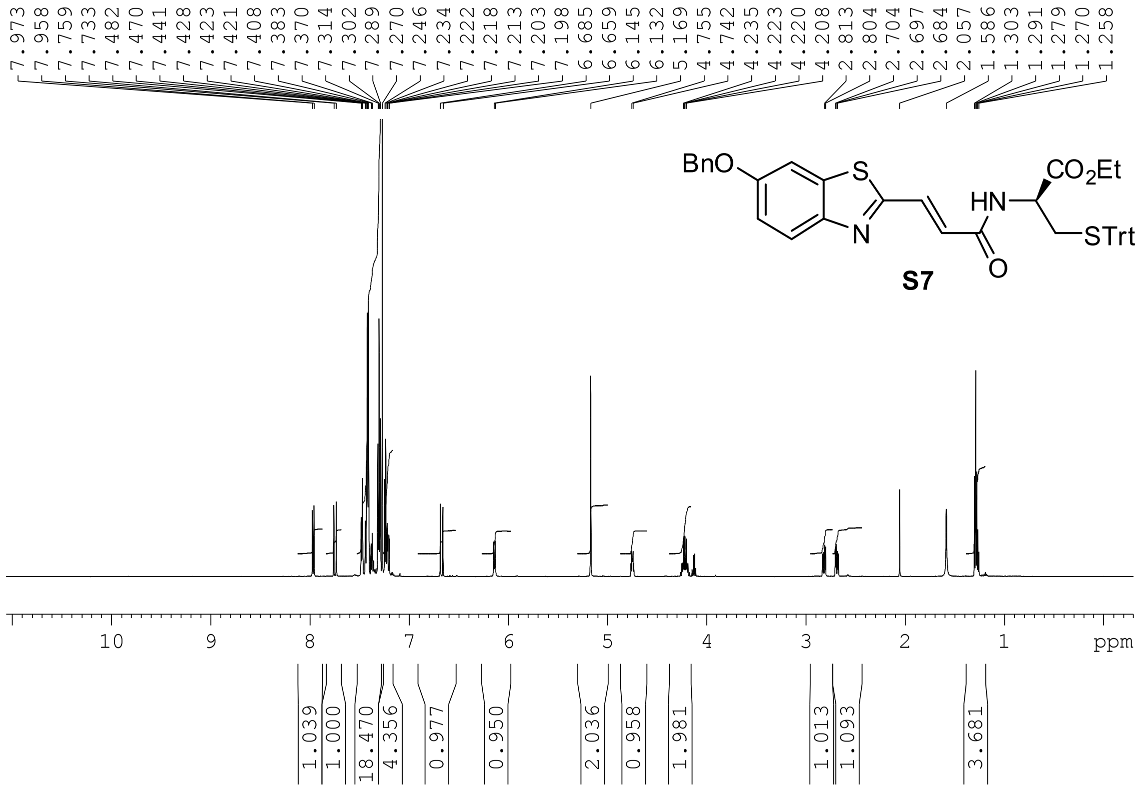

^{13}C NMR spectrum of **S7** ($CDCl_3$)

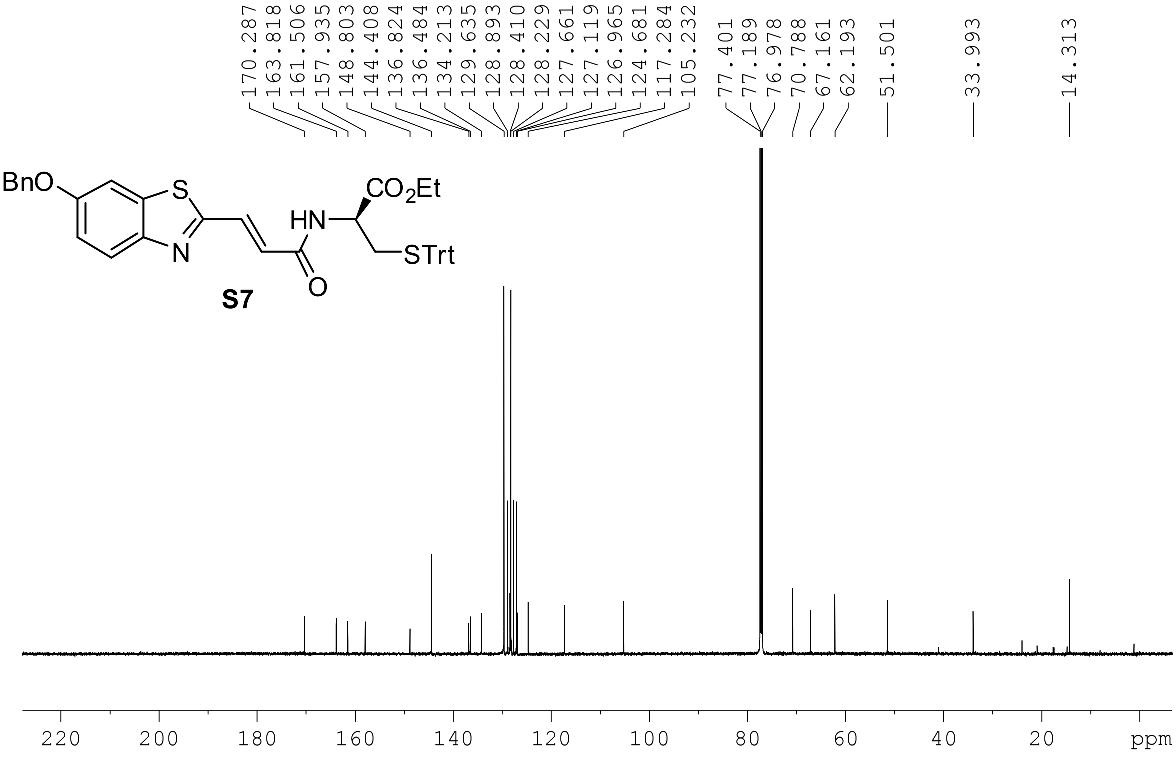

^{1}H NMR spectrum of **S8** ($CDCl_3$)

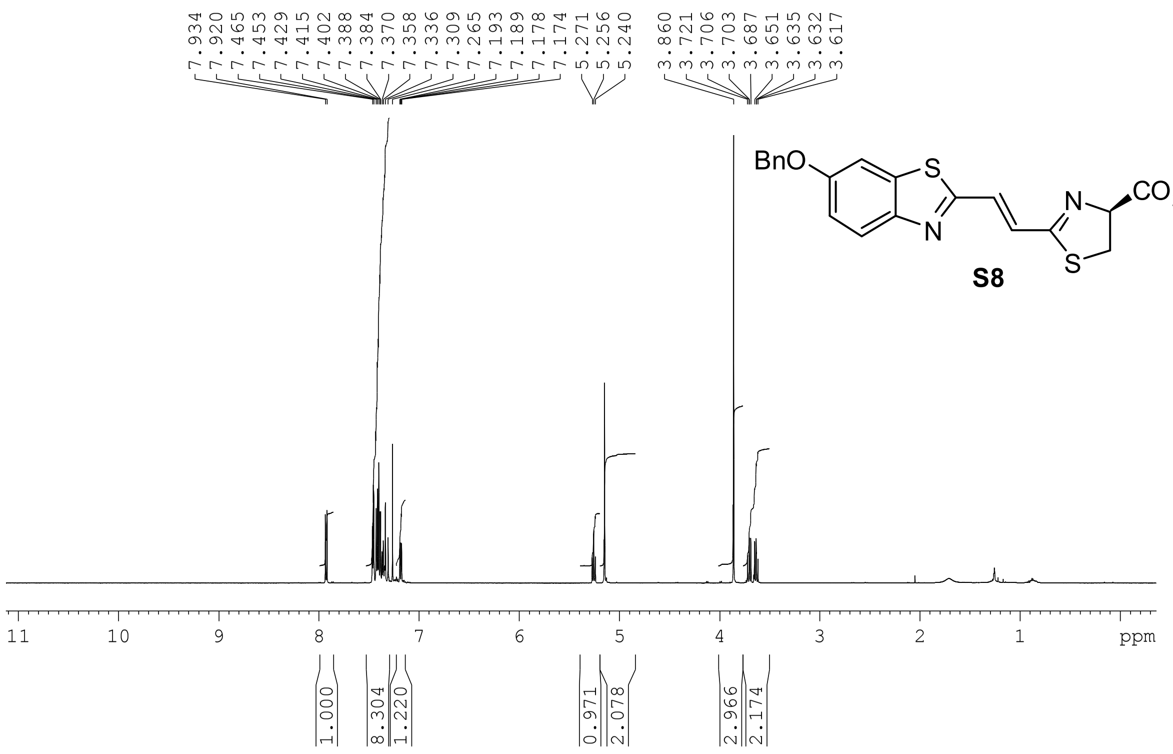

^{13}C NMR spectrum of **S8** ($CDCl_3$)

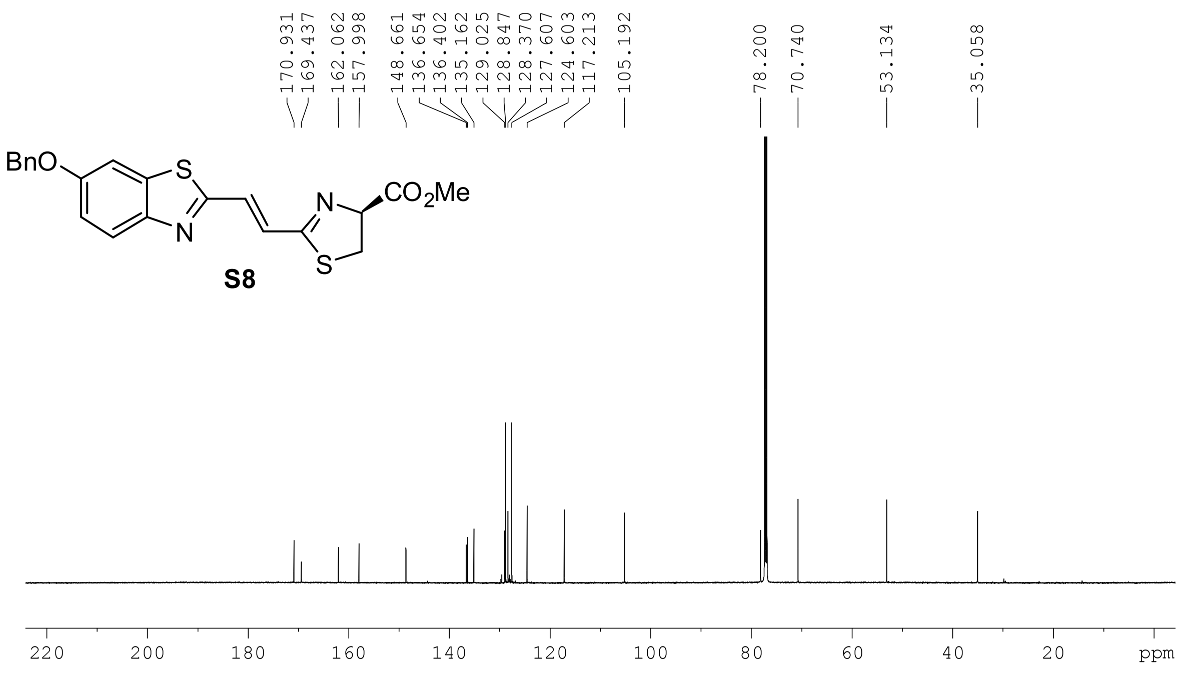

^1H NMR spectrum of **S9** ($CDCl_3$)

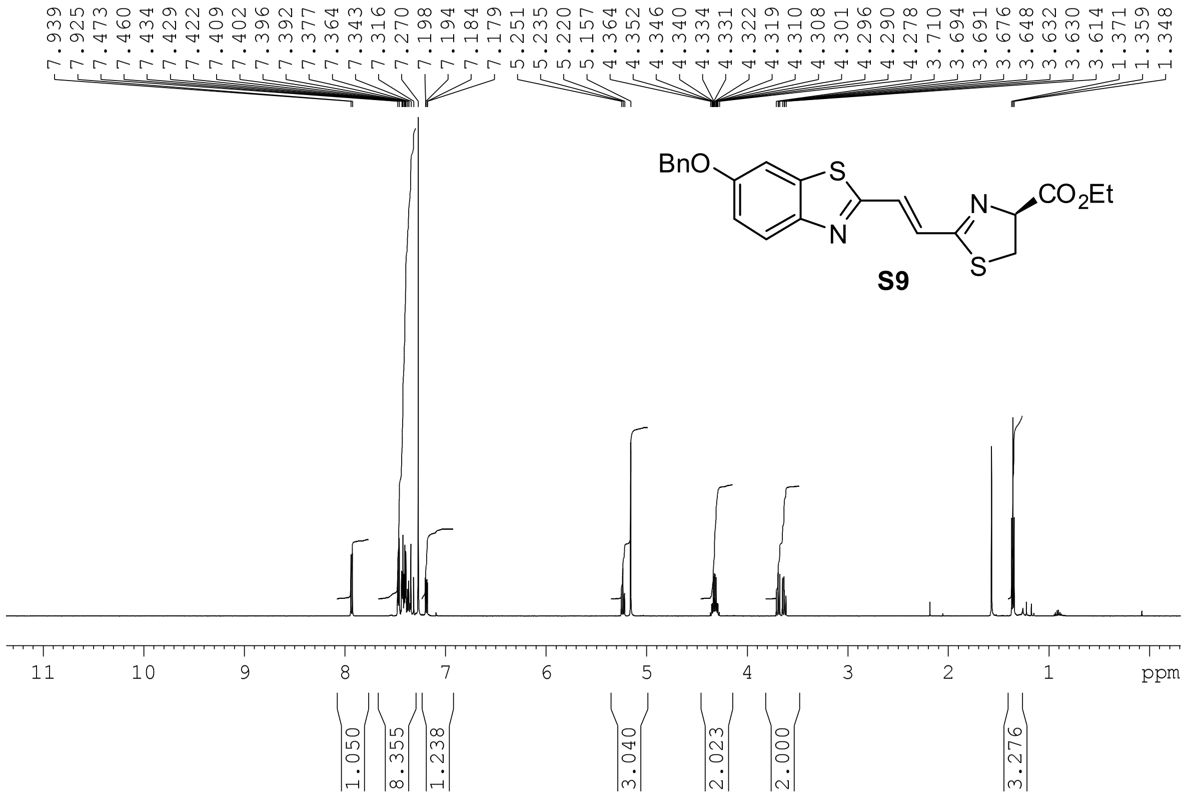

^{13}C NMR spectrum of **S9** ($CDCl_3$)

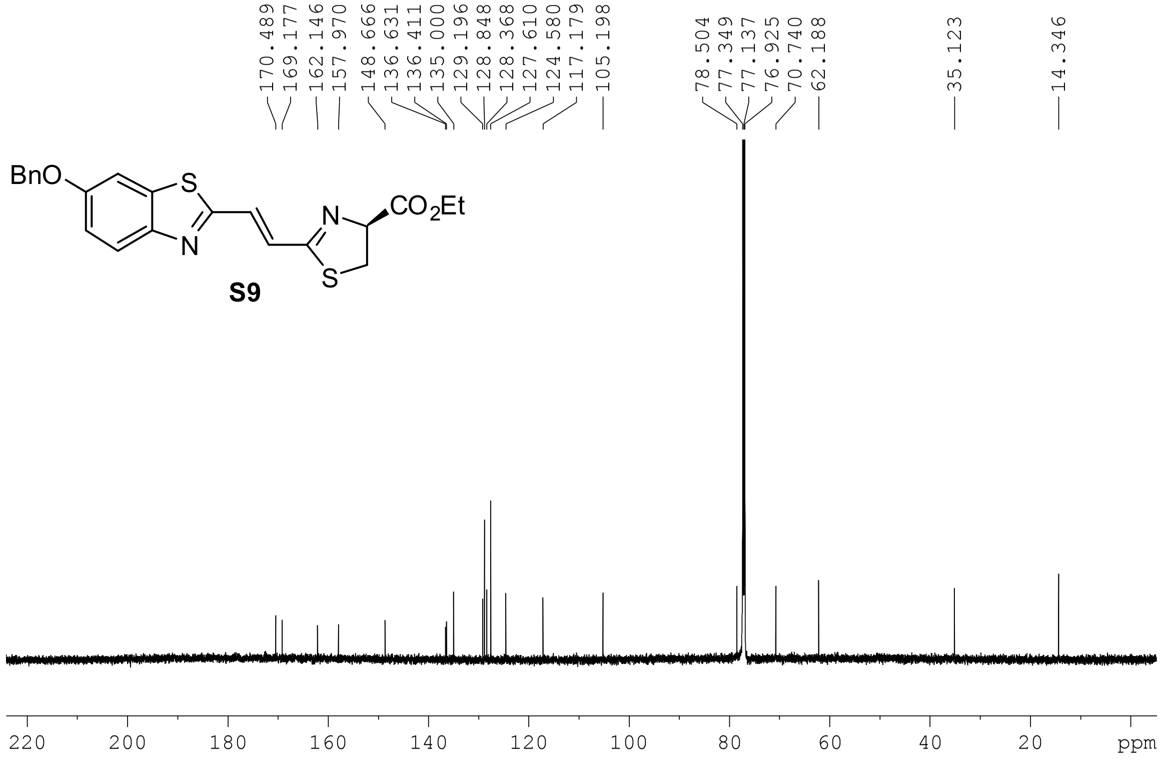

^{1}H NMR spectrum of **S10** (MeOD)

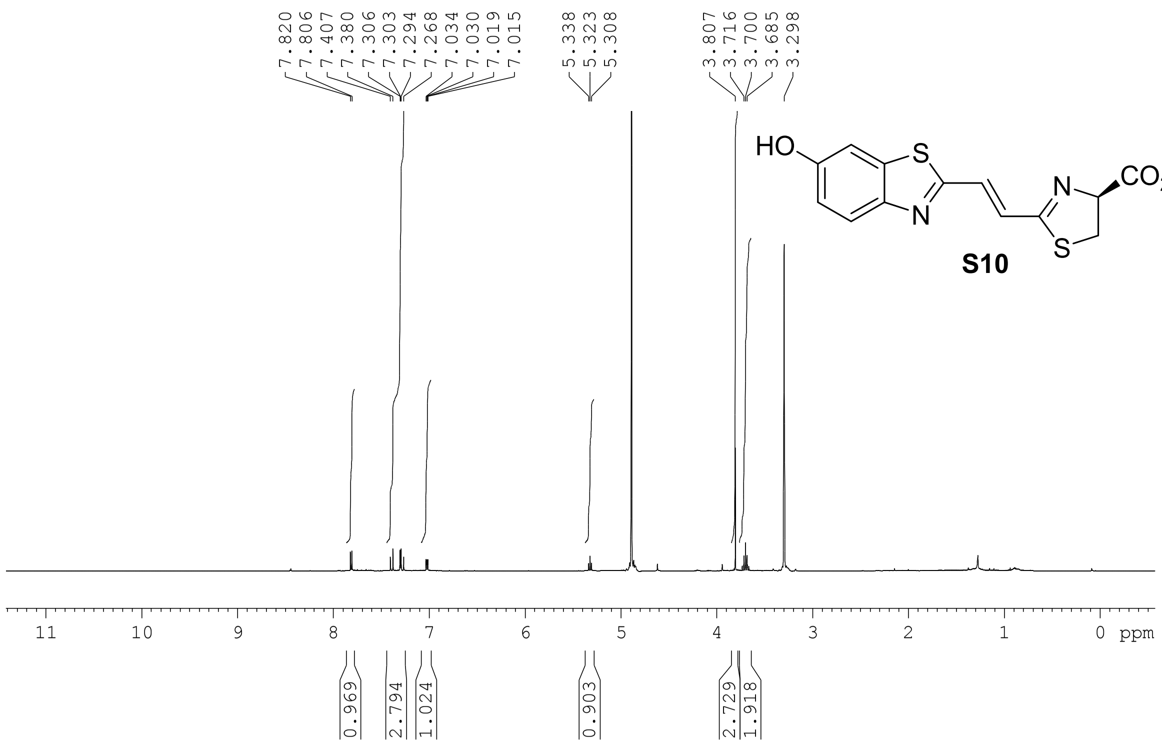

^{13}C NMR spectrum of **S10** (MeOD)

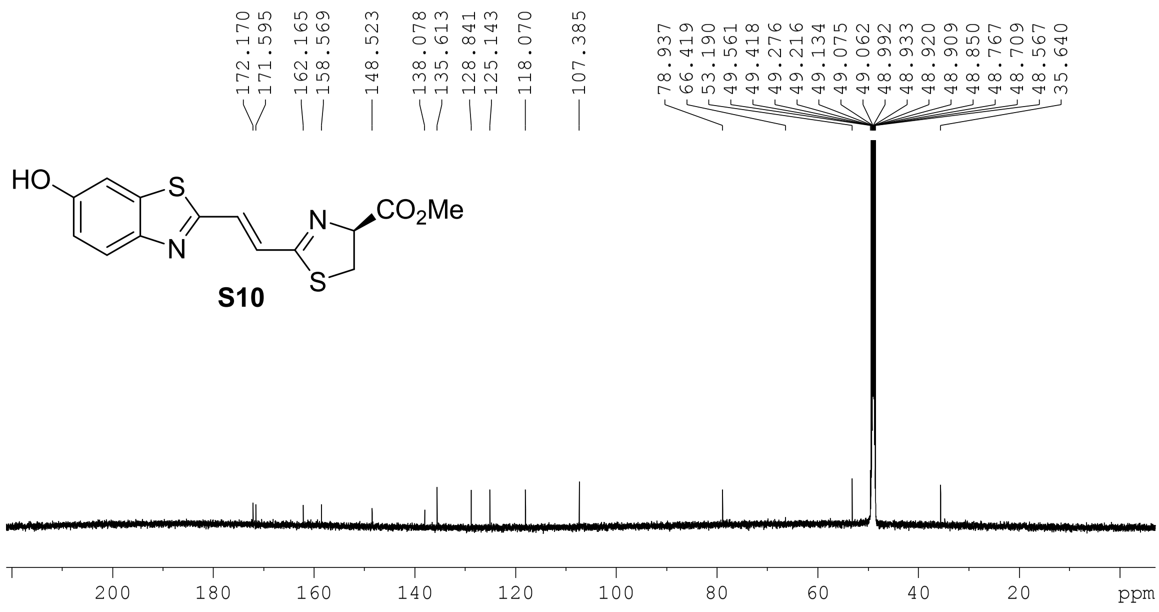

^{1}H NMR spectrum of **S11** ($CDCl_3$)

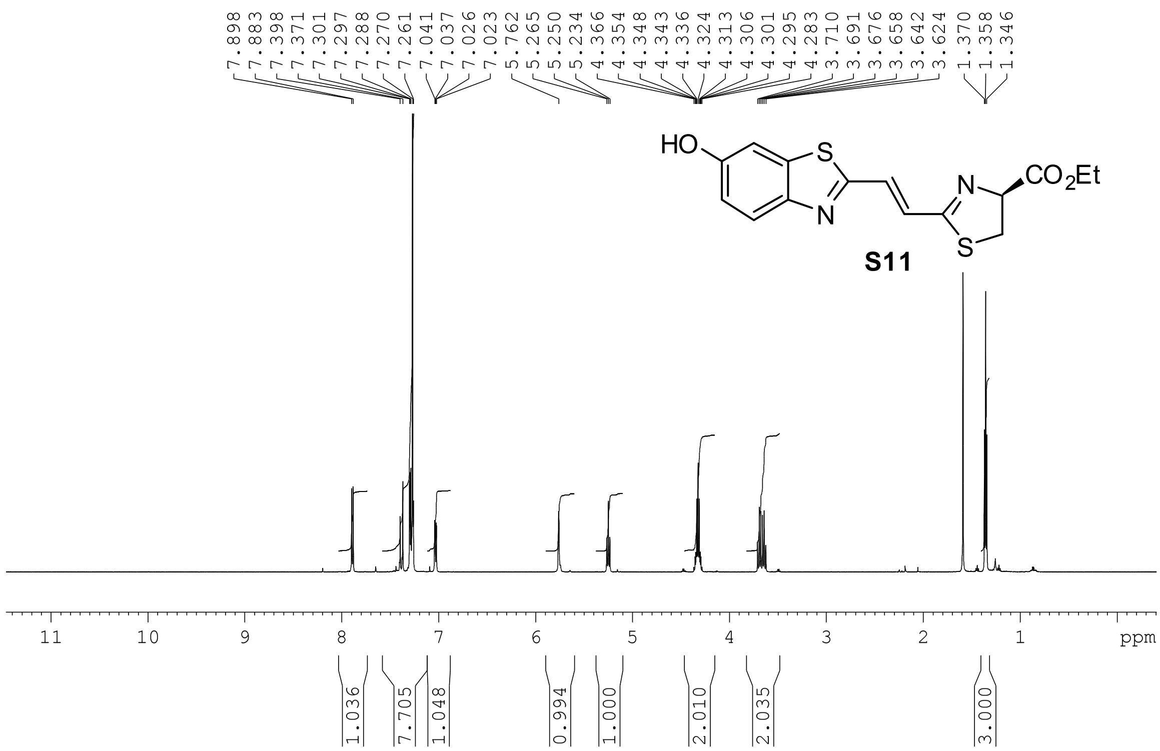

^{13}C NMR spectrum of **S11** ($CDCl_3$)

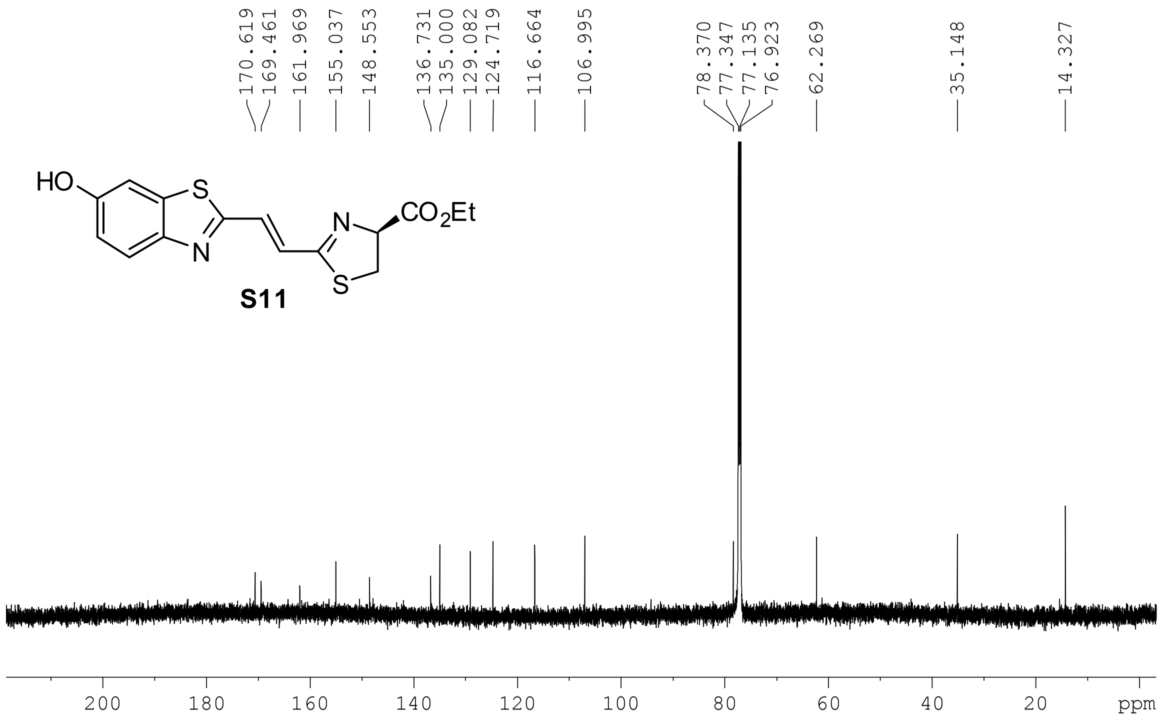

Supplementary References.

[1] F. F. Craig, A. C. Simmonds, D. Watmore, F. McCapra, M. R. White, *Biochem. J.* **1991**, *276*, 637-641.

[2] A. Cornish-Bowden, *Basic Mathematics foe Biochemists*, Oxford University Press, USA, **1999**.

[3] K. C. Straathof, M. A. Pule, P. Yotnda, G. Dotti, E. F. Vanin, M. K. Brenner, H. E. Heslop, D. M. Spencer, C. M. Rooney, *Blood* **2005**, *105*, 4247-4254.

[4] A. A. Akwabi-Ameyaw, D. N. Deaton, R. B. Mcfadyen, F. Navas, X. Farnesoid, WO Patent 2009005998 (A1) filed 13 June 2008, and issued 8 Jan. 2009.

[5] P. J. Crowley, R. Salmon, O. A. Sageot, D. P. Bacon, D. W. Langford, WO Patent 2004108663 (A1) filed 28 June 2004, and issued 16 Dec. 2004.

[6] J. Rudolph, H. Theis, R. Hanke, R. Endermann, L. Johannsen, F. Geschke, *J. Med. Chem.* **2001**, *44*, 619-626.

[7] J. R. Mays, J. A. Restituyo, R. J. Katzenberger, D. A. Wassarman, S. R. Rajski, *Tetrahedron Lett.* **2007**, *48*, 4579-4583.
